# Supplementary material for: Genome-Wide Identification of Brassicaceae Hormone-Related Transcription Factors and Their Roles in Stress Adaptation and Plant Height Regulation in Allotetraploid Rapeseed
Source: Int J Mol Sci. 2022 Aug 6;23(15):8762. doi: 10.3390/ijms23158762 (PMC9369146; doi:10.3390/ijms23158762)
Supplement: Supplementary file 1 [file ijms-23-08762-s001.zip › Figure S1.pdf]

Supplemental Figure S1. Number of phytohormone-related *TFs* among different species.

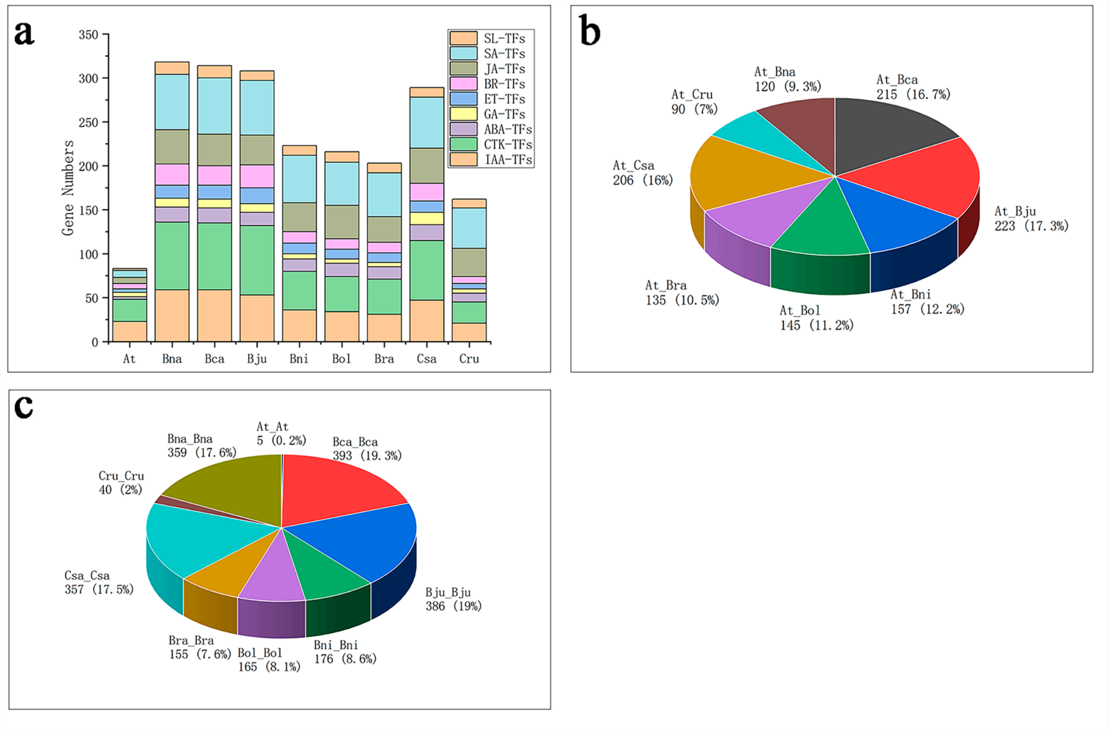

a Number of phytohormone-related *TFs* in Brassicaceae species. At, *Arabidopsis thaliana*; Bna, *Brassica napus*; Bca, *Brassica carinata*; Bju, *Brassica juncea*; Bni, *Brassica nigra*; Bol, *Brassica oleracea*; Bra, *Brassica rapa*; Csa, *Camelina sativa*; Cru, *Capsella rubella*. **b** Percent of phytohormone-related *TFs* pairs between *Arabidopsis* and other Brassicaceae species; **c** Percent of phytohormone-related *TFs* pairs in each Brassicaceae species.
