# Supplementary material for: Genome-Wide Identification of Brassicaceae Hormone-Related Transcription Factors and Their Roles in Stress Adaptation and Plant Height Regulation in Allotetraploid Rapeseed
Source: Int J Mol Sci. 2022 Aug 6;23(15):8762. doi: 10.3390/ijms23158762 (PMC9369146; doi:10.3390/ijms23158762)

Supplemental Figure S2. Chromosomal location of Brassicaceae phytohormone-related *TFs*.

Fig. S2-1 The location of *Arabidopsis* hormone *TFs* on chromosomes.

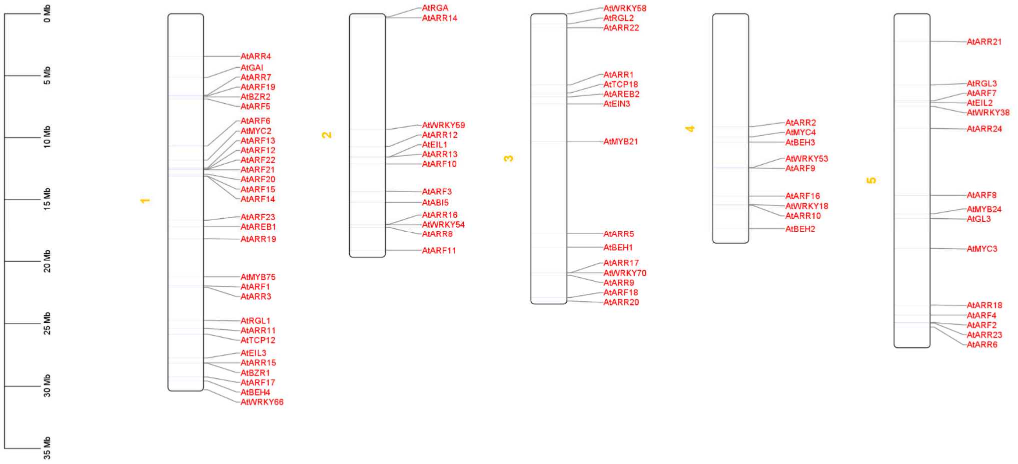

Fig. S2-2 The location of *Brassica napus* hormone *TFs* on chromosomes.

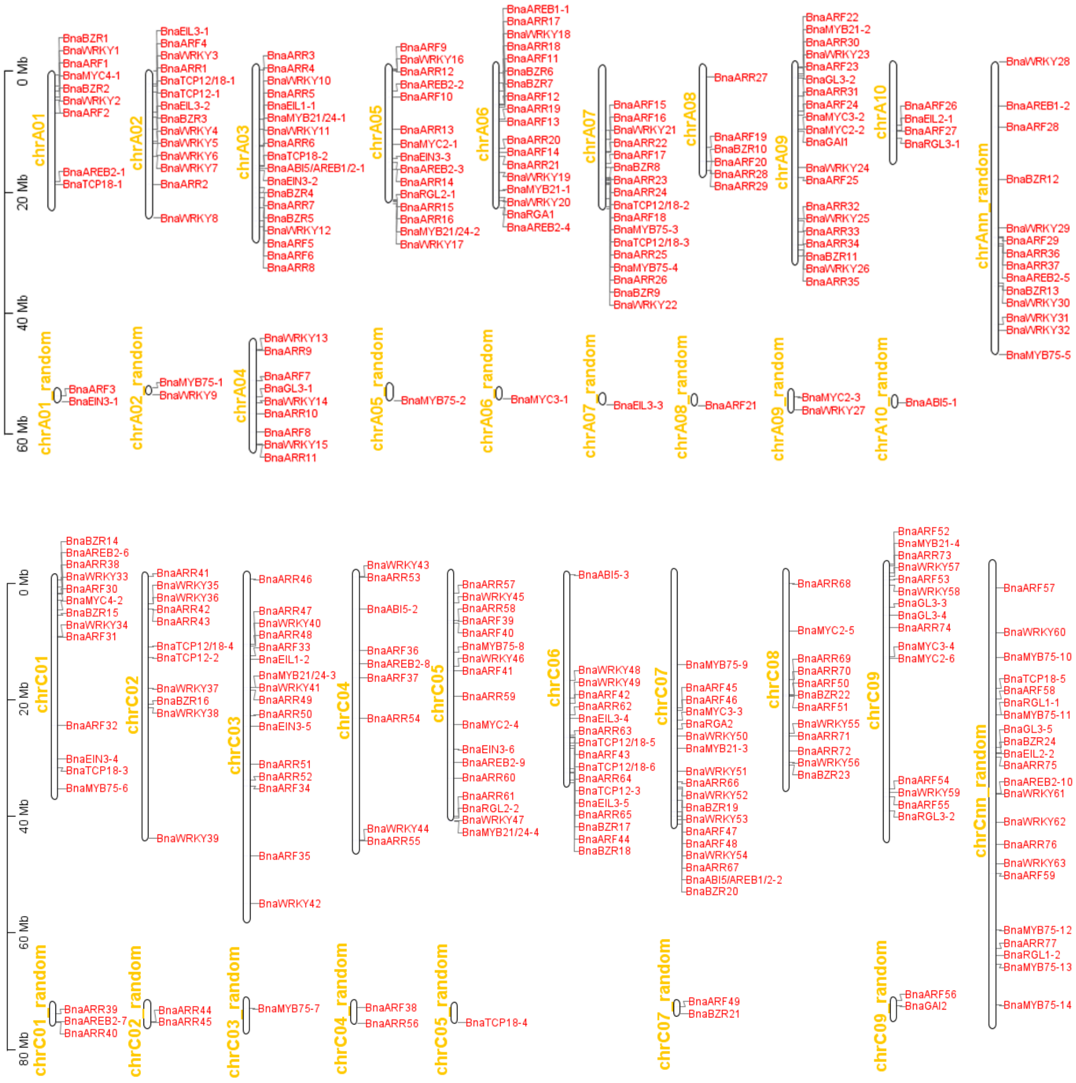

Fig. S2-3 The location of *Brassica carinata* hormone *TFs* on chromosomes.

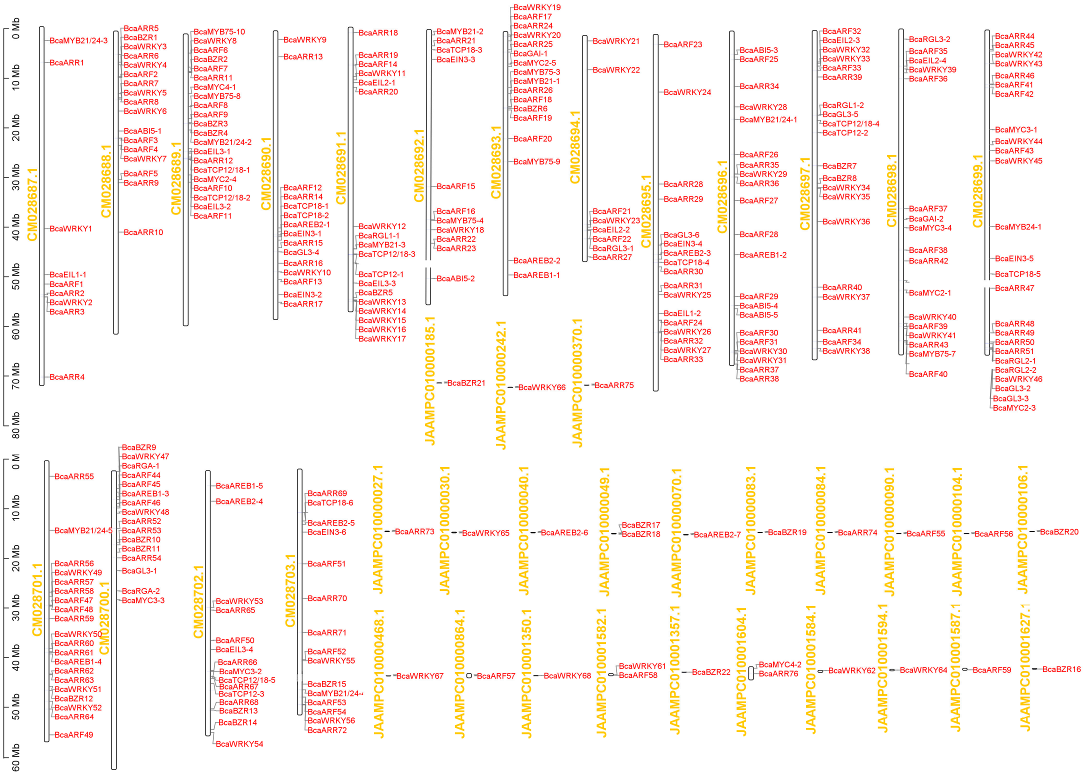

Fig. S2-4 The location of *Brassica juncea* hormone *TFs* on chromosomes.

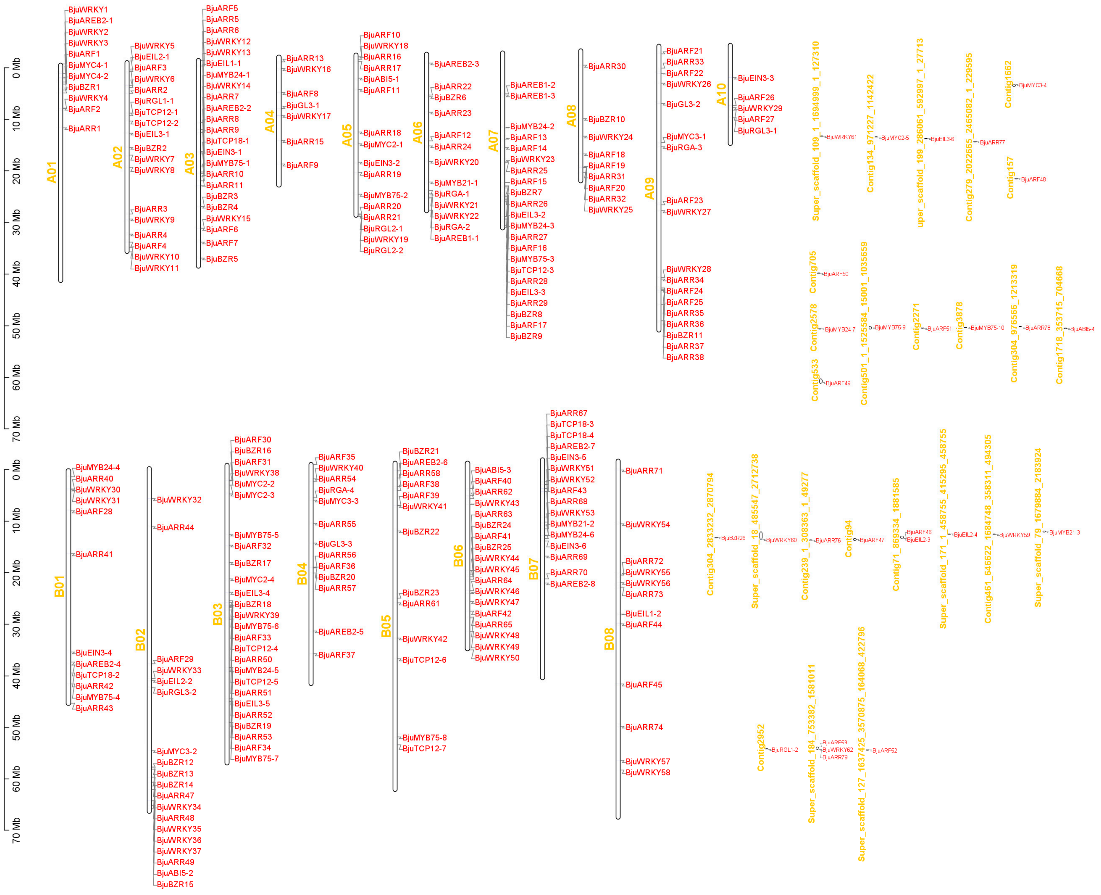

Fig. S2-5 The location of *Brassica nigra* hormone *TFs* on chromosomes.

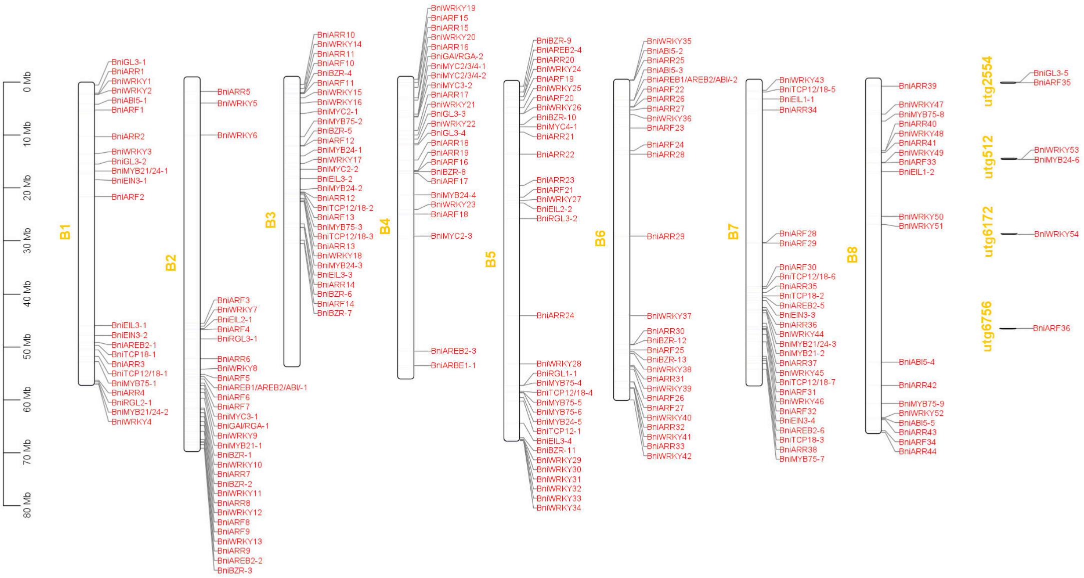

Fig. S2-6 The location of *Brassica oleracea* hormone *TFs* on chromosomes.

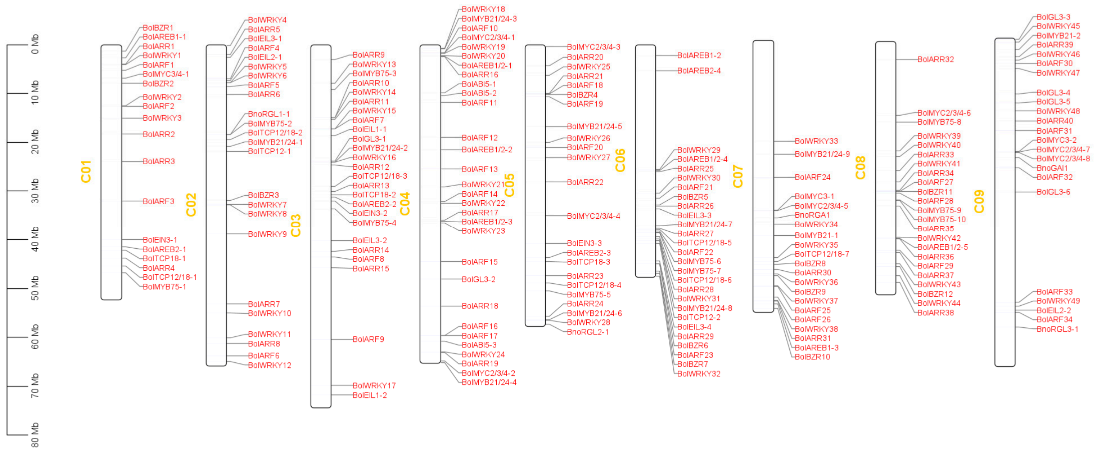

Fig. S2-7 The location of *Brassica rapa* hormone *TFs* on chromosomes.

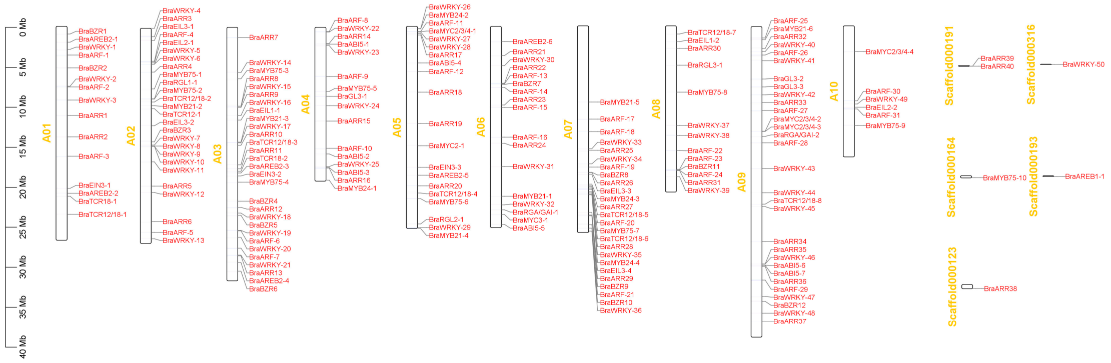

Fig. S2-8 The location of *Camelina sativa* hormone *TFs* on chromosomes.

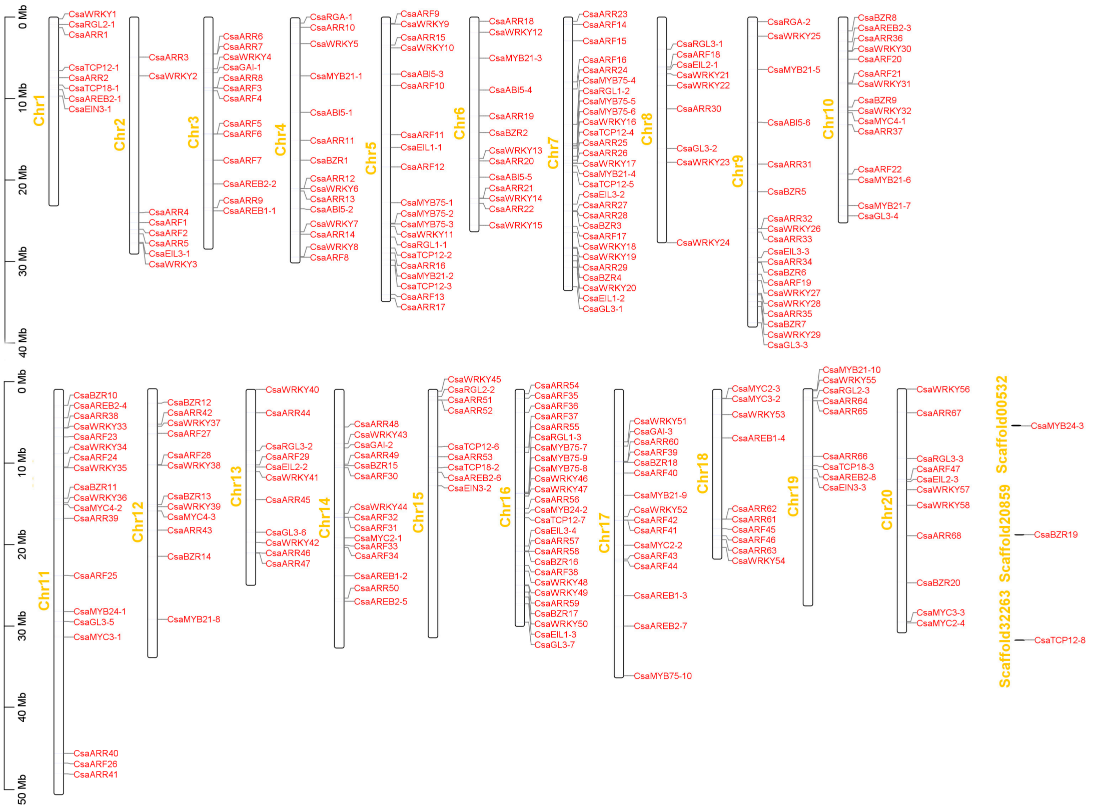

Fig. S2-9 The location of *Capsella rubella* hormone *TFs* on chromosomes.

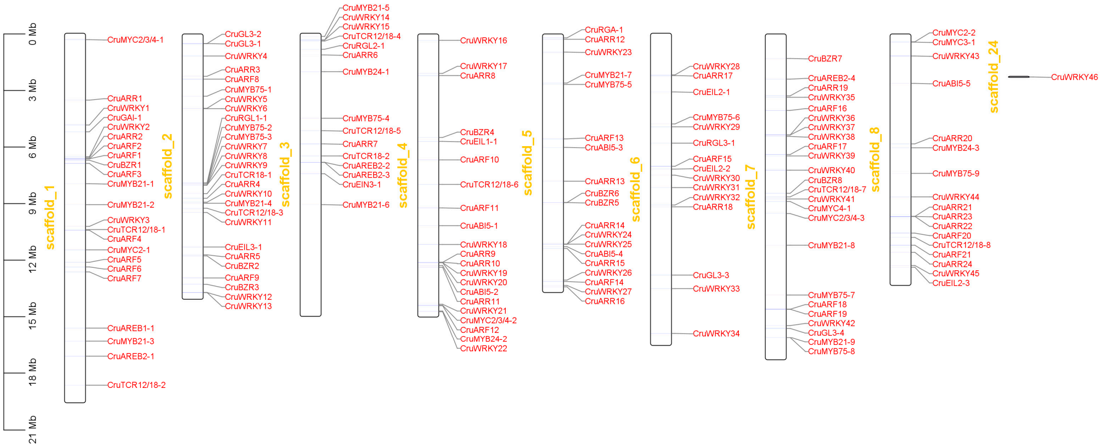

Supplement: Supplementary file 1 [file ijms-23-08762-s001.zip › Figure S2.pdf]
