# Supplementary material for: Genome-Wide Identification of Brassicaceae Hormone-Related Transcription Factors and Their Roles in Stress Adaptation and Plant Height Regulation in Allotetraploid Rapeseed
Source: Int J Mol Sci. 2022 Aug 6;23(15):8762. doi: 10.3390/ijms23158762 (PMC9369146; doi:10.3390/ijms23158762)

Supplemental Figure S3. Conserved domains and gene structure of Brassicaceae hormone-related TFs genes.

Figure. S3-1 Conserved domains and gene structure analysis in Brassicaceae IAA-related TFs genes.

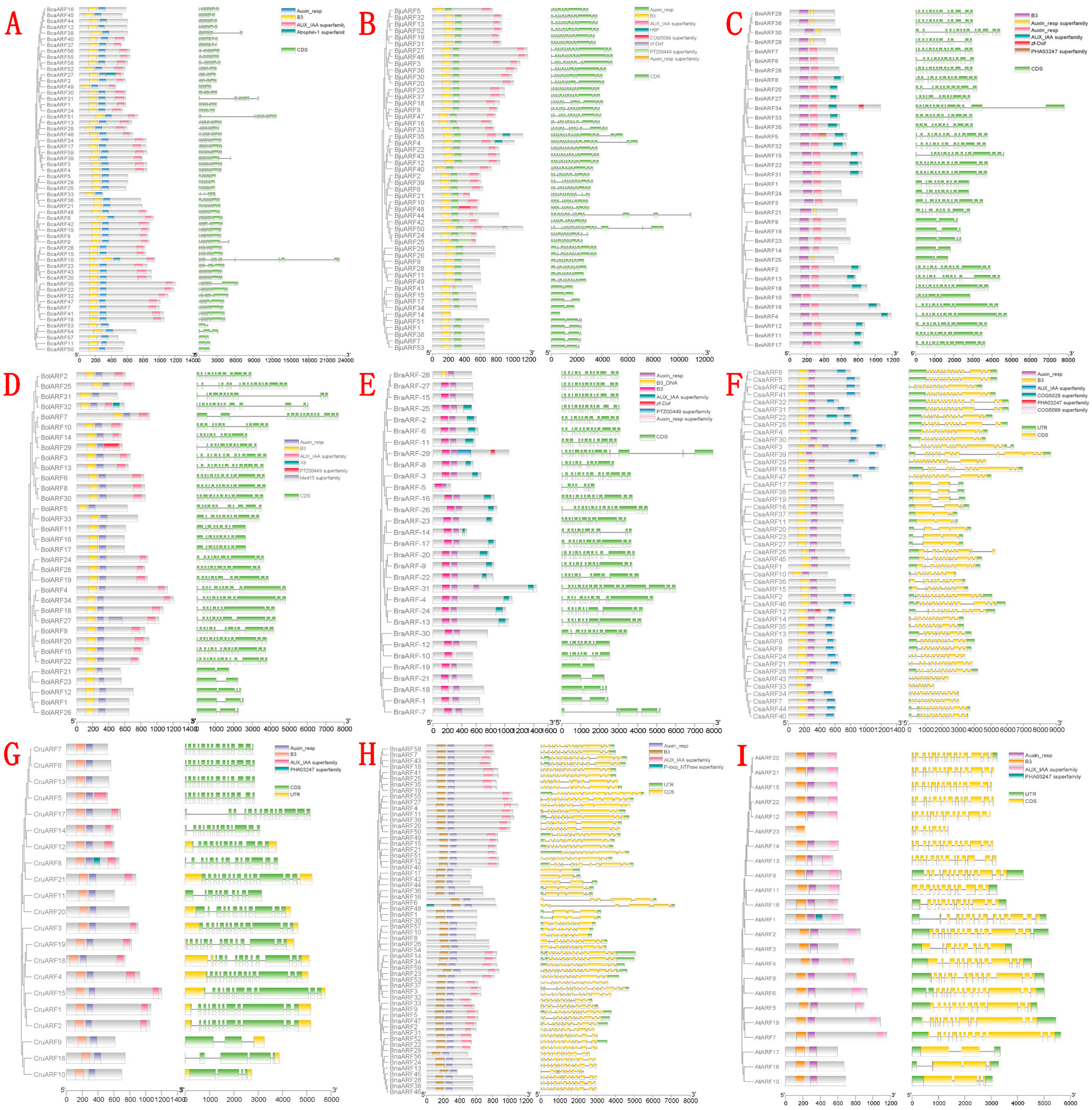

Figure. S3-2 Conserved domains and gene structure analysis in Brassicaceae CTK-related TFs genes.

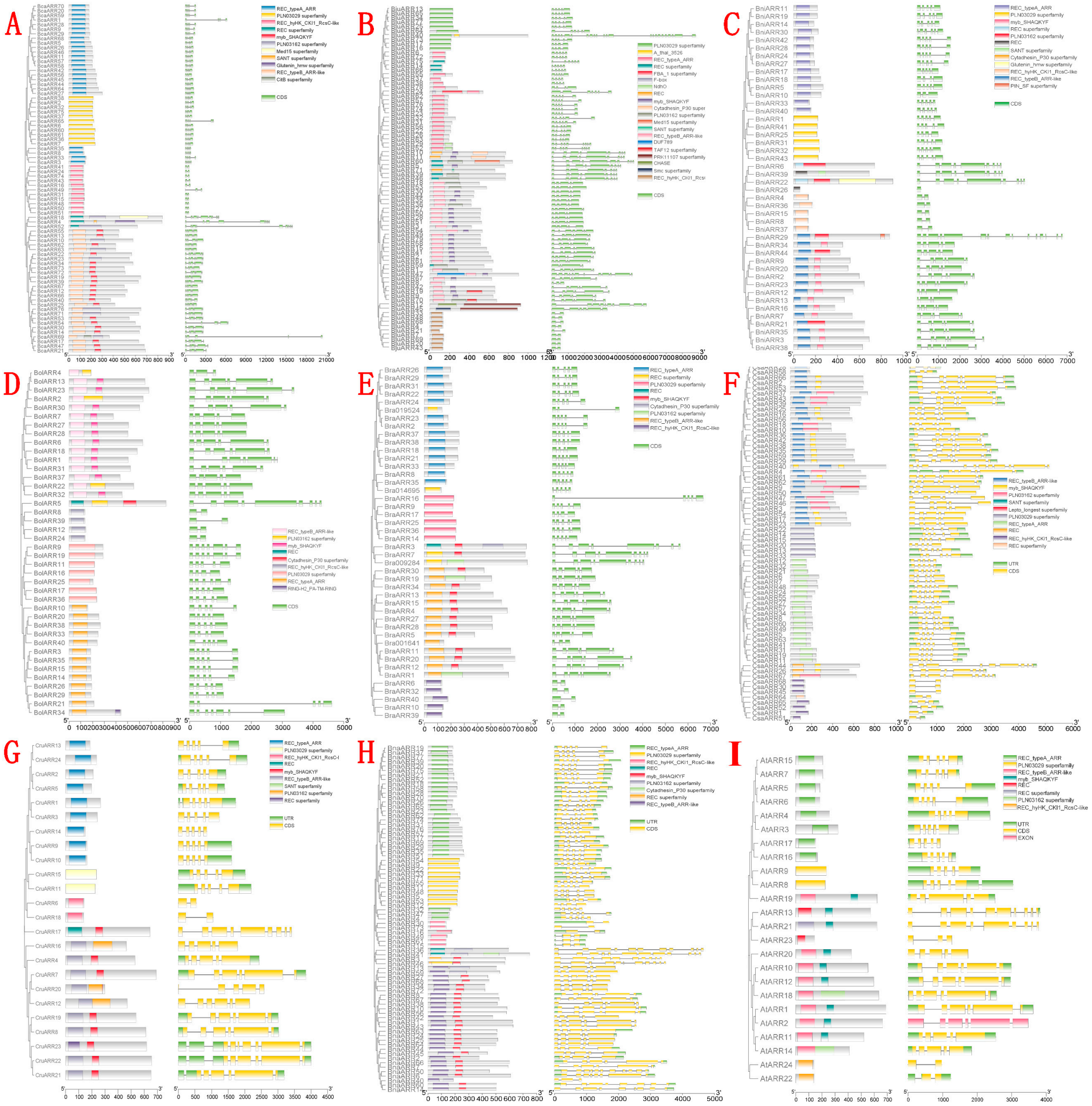

Figure. S3-3 Conserved domains and gene structure analysis in Brassicaceae ABA-related TFs genes.

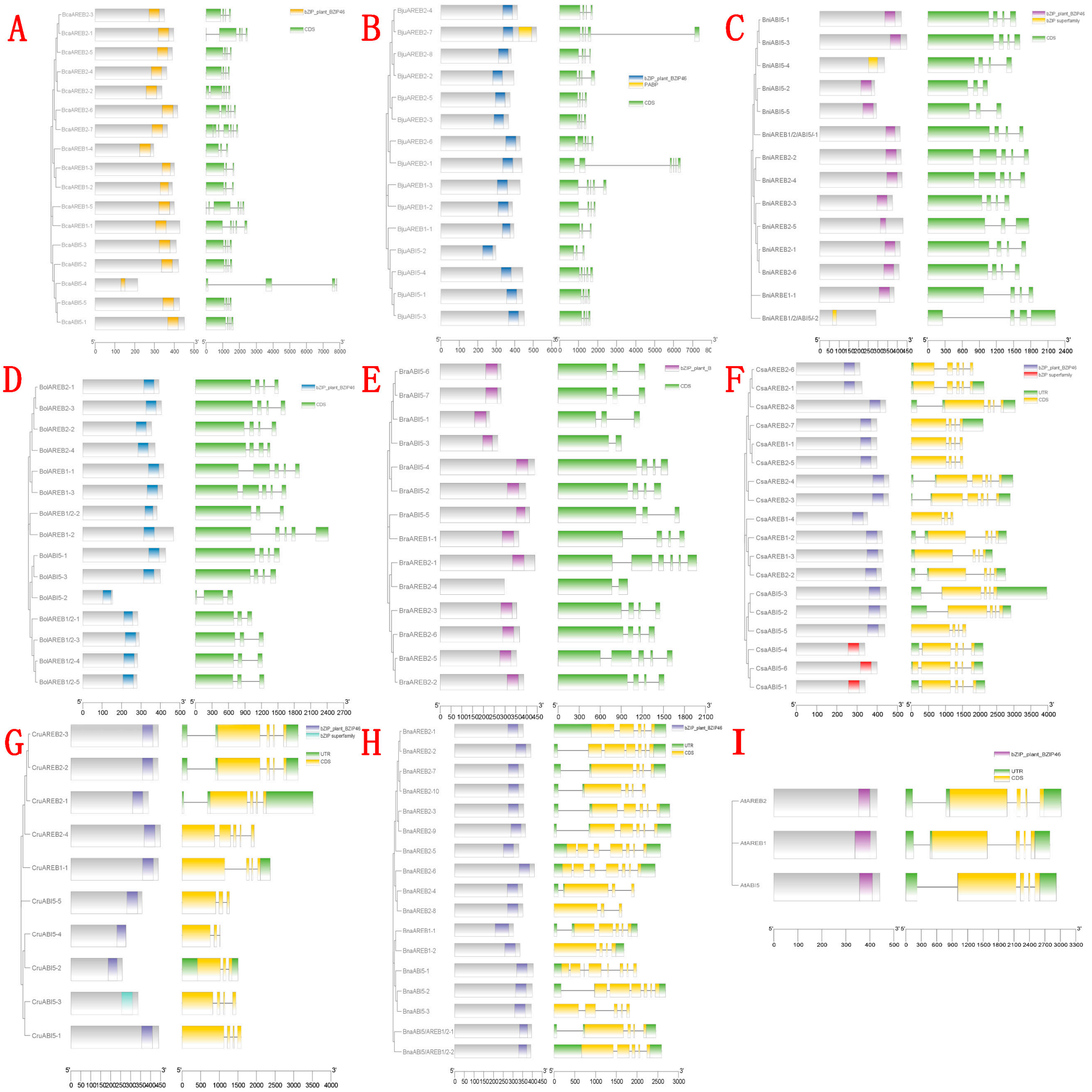

Figure. S3-4 Conserved domains and gene structure analysis in Brassicaceae GA-related TFs genes.

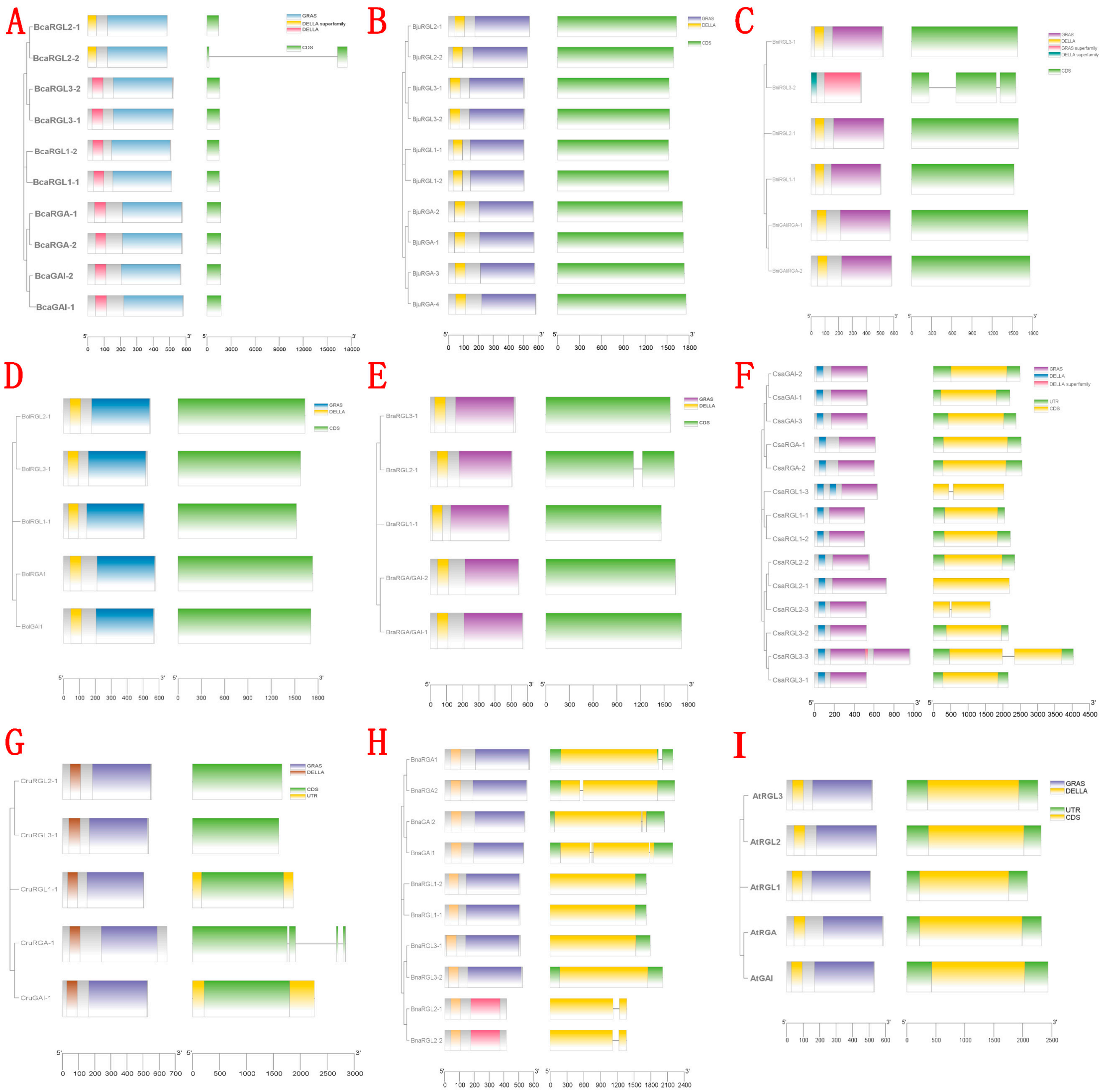

Figure. S3-5 Conserved domains and gene structure analysis in Brassicaceae ET-related TFs genes.

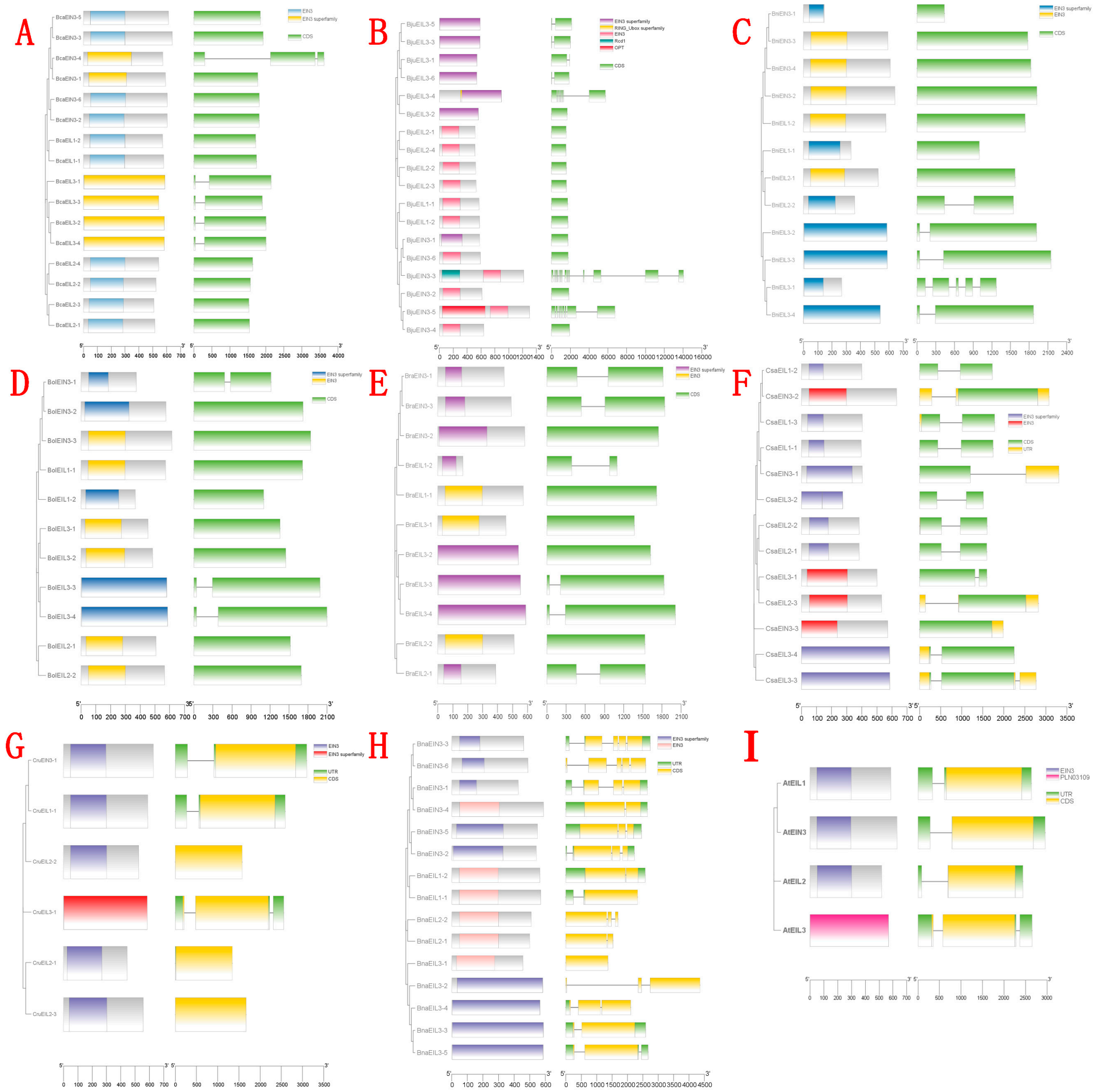

Figure. S3-6 Conserved domains and gene structure analysis in Brassicaceae BR-related TFs genes.

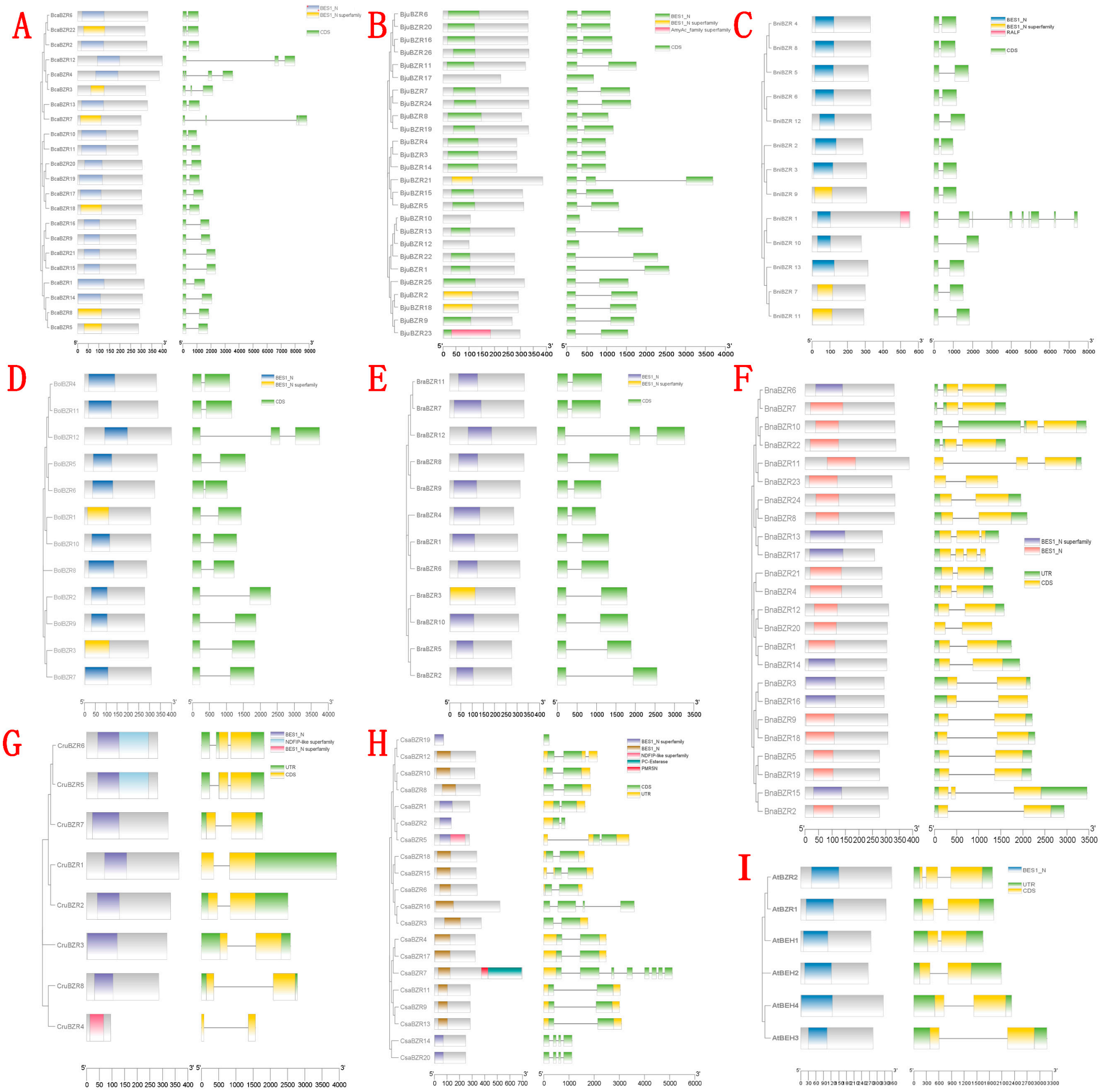

Figure. S3-7 Conserved domains and gene structure analysis in Brassicaceae JA-related TFs genes.

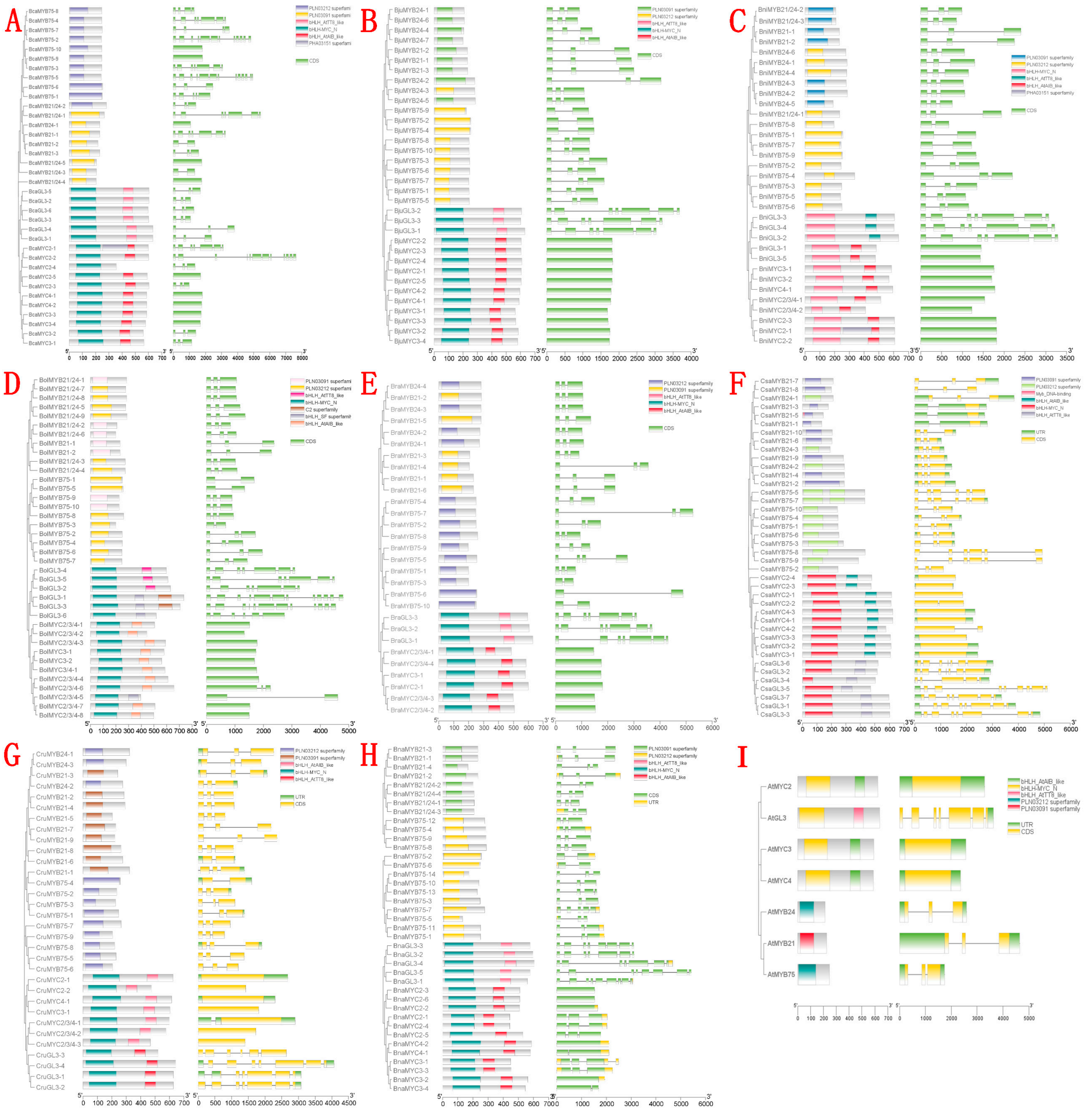

Figure. S3-8 Conserved domains and gene structure analysis in Brassicaceae SA-related TFs genes.

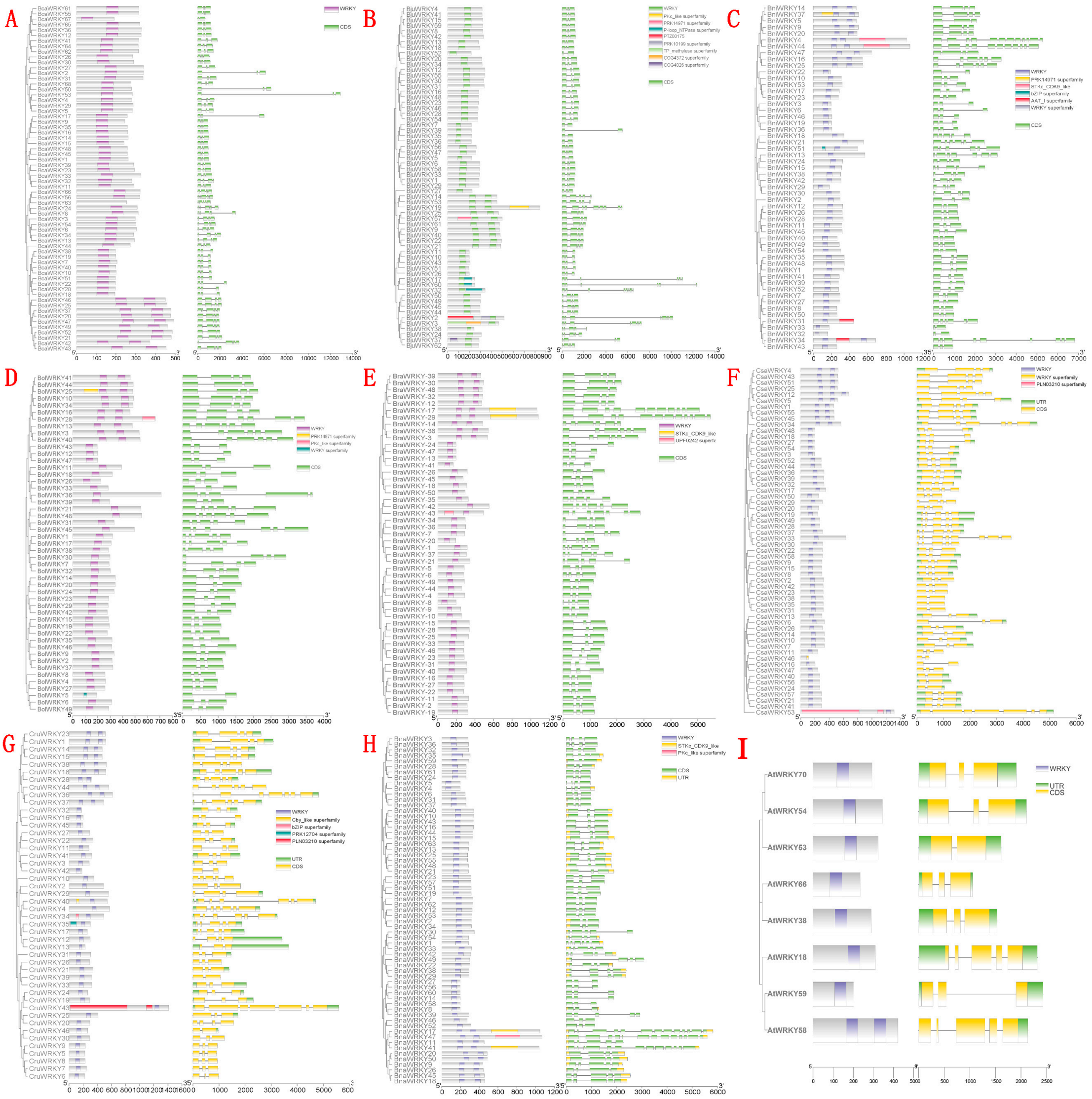

Figure. S3-9 Conserved domains and gene structure analysis in Brassicaceae SL-related TFs genes.

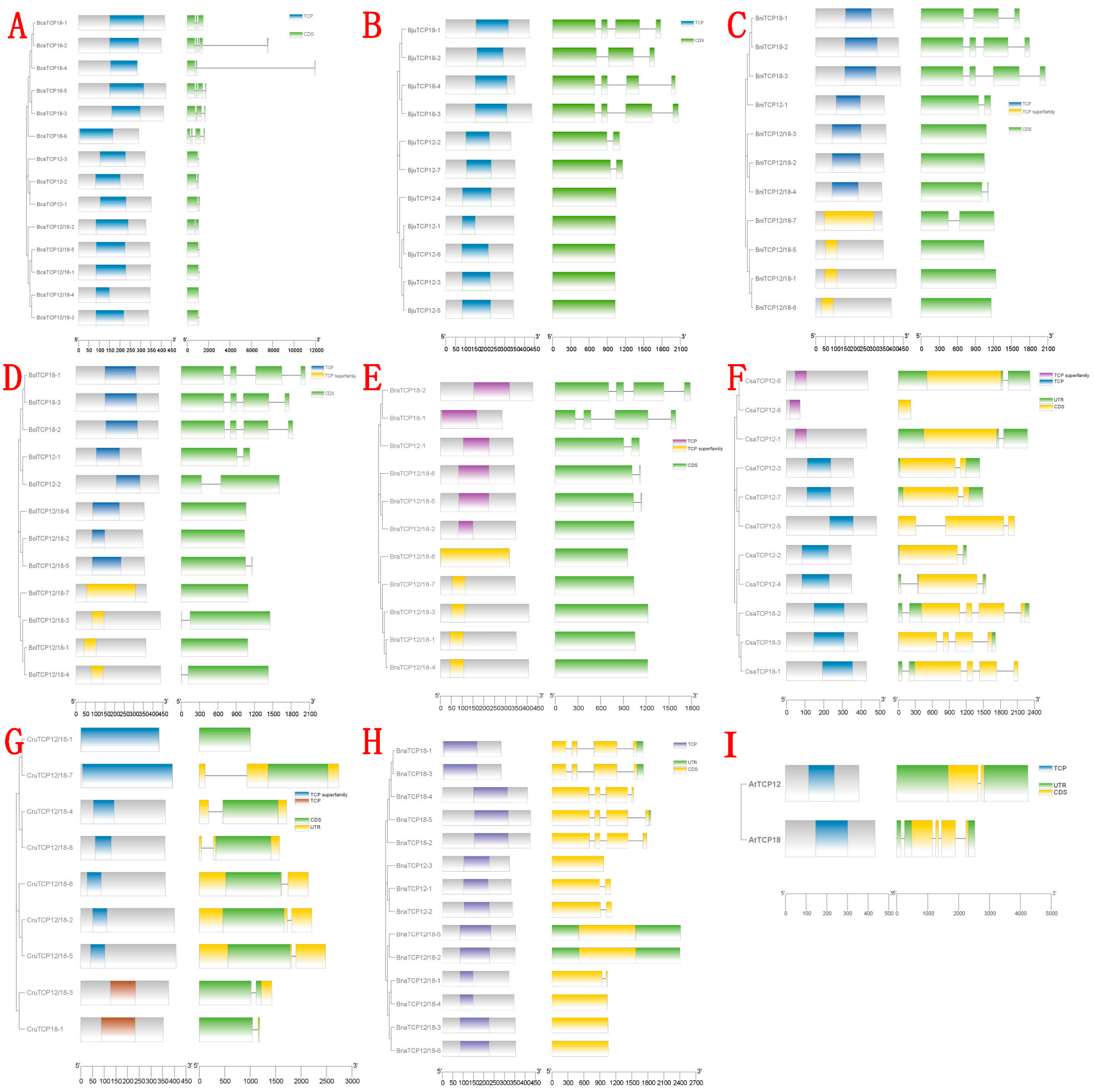

Supplement: Supplementary file 1 [file ijms-23-08762-s001.zip › Figure S3.pdf]
