# Supplementary material for: Genome-Wide Identification of Brassicaceae Hormone-Related Transcription Factors and Their Roles in Stress Adaptation and Plant Height Regulation in Allotetraploid Rapeseed
Source: Int J Mol Sci. 2022 Aug 6;23(15):8762. doi: 10.3390/ijms23158762 (PMC9369146; doi:10.3390/ijms23158762)

Supplemental Figure S4. Phylogenetic analysis of Brassicaceae hormone-related TFs.

Figure. S4-1 Phylogenetic analysis of Brassicaceae IAA-related TFs.

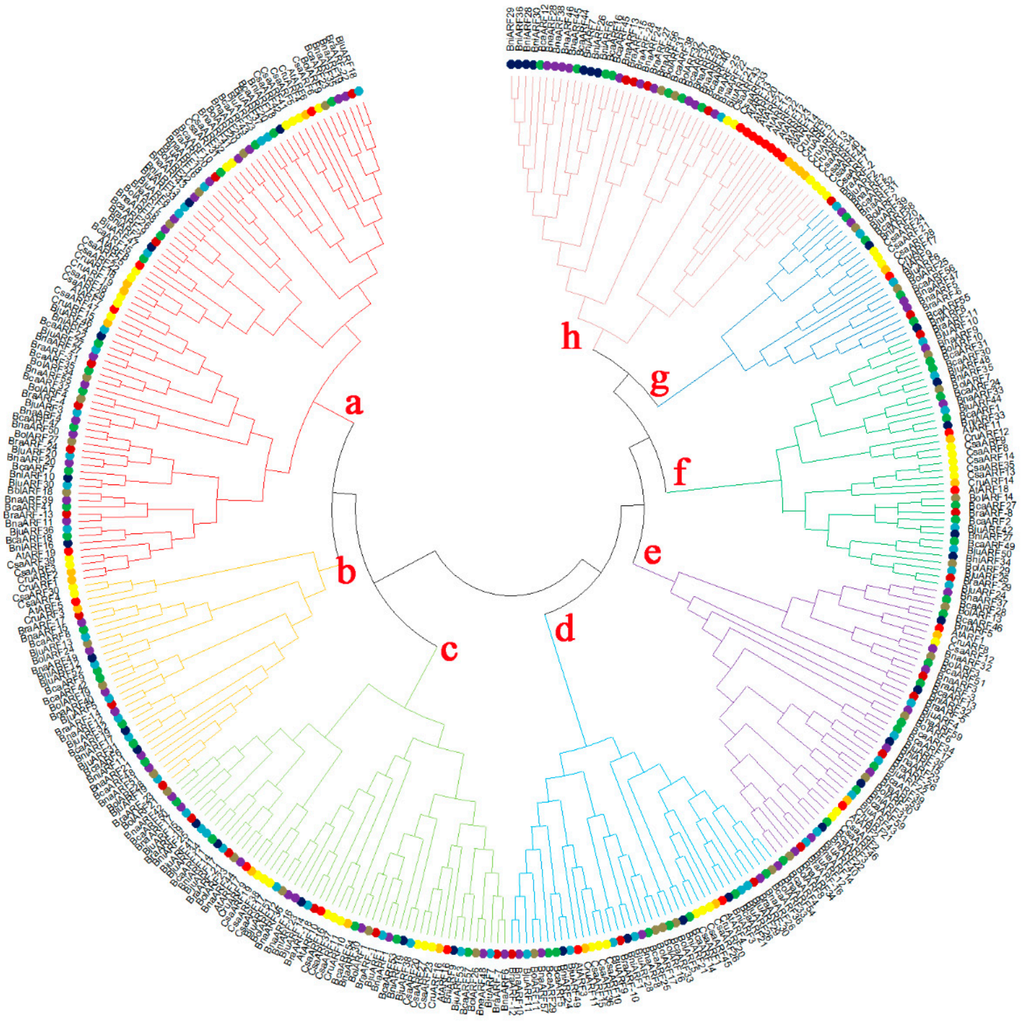

Figure. S4-2 Phylogenetic analysis of Brassicaceae CTK-related TFs.

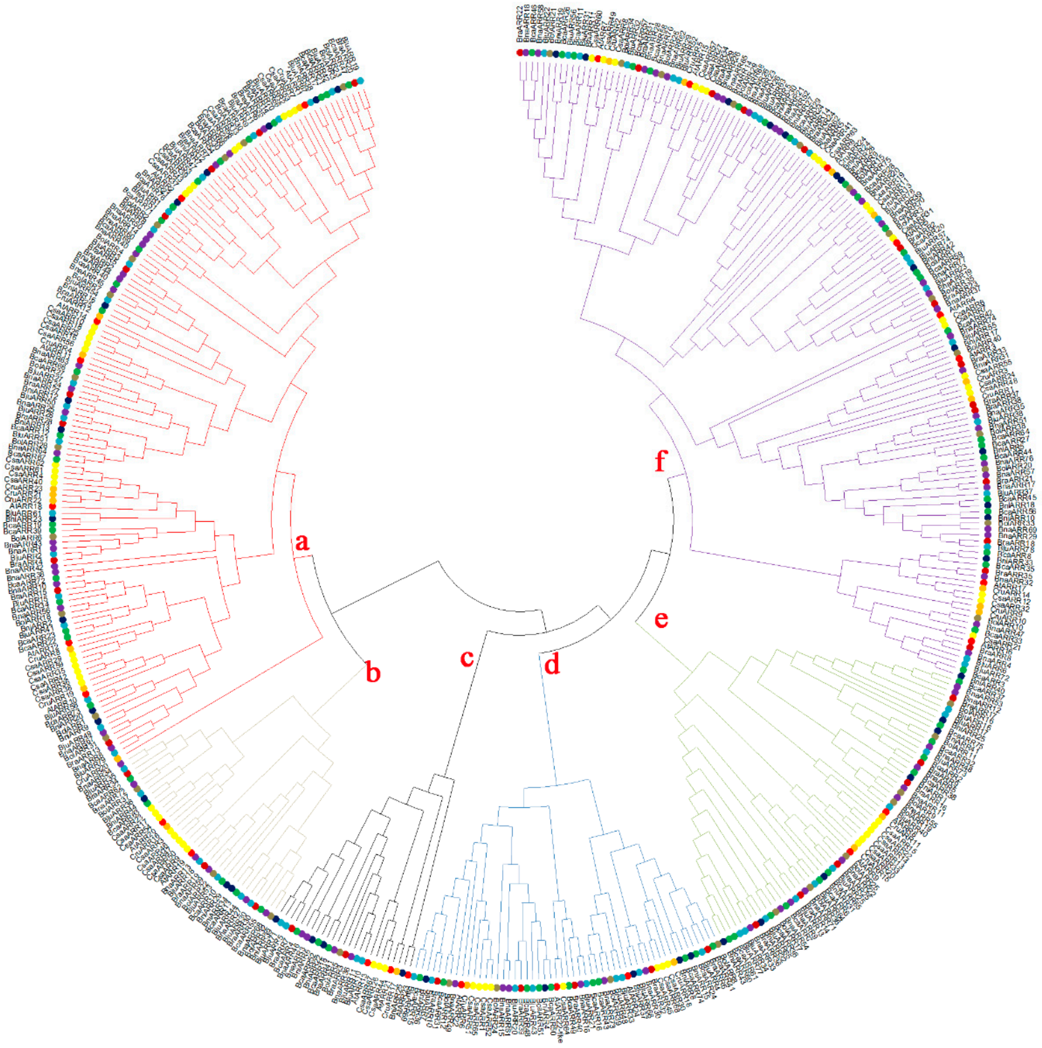

Figure. S4-3 Phylogenetic analysis of Brassicaceae ABA-related TFs.

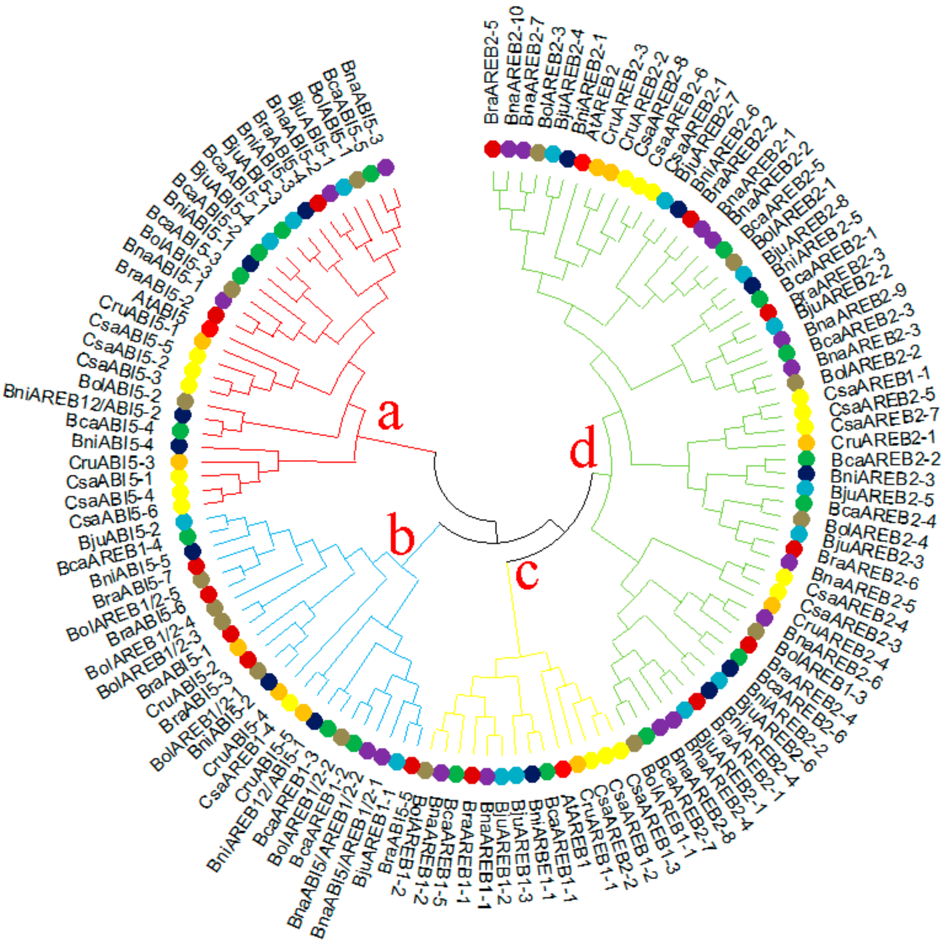

Figure. S4-4 Phylogenetic analysis of Brassicaceae GA-related TFs.

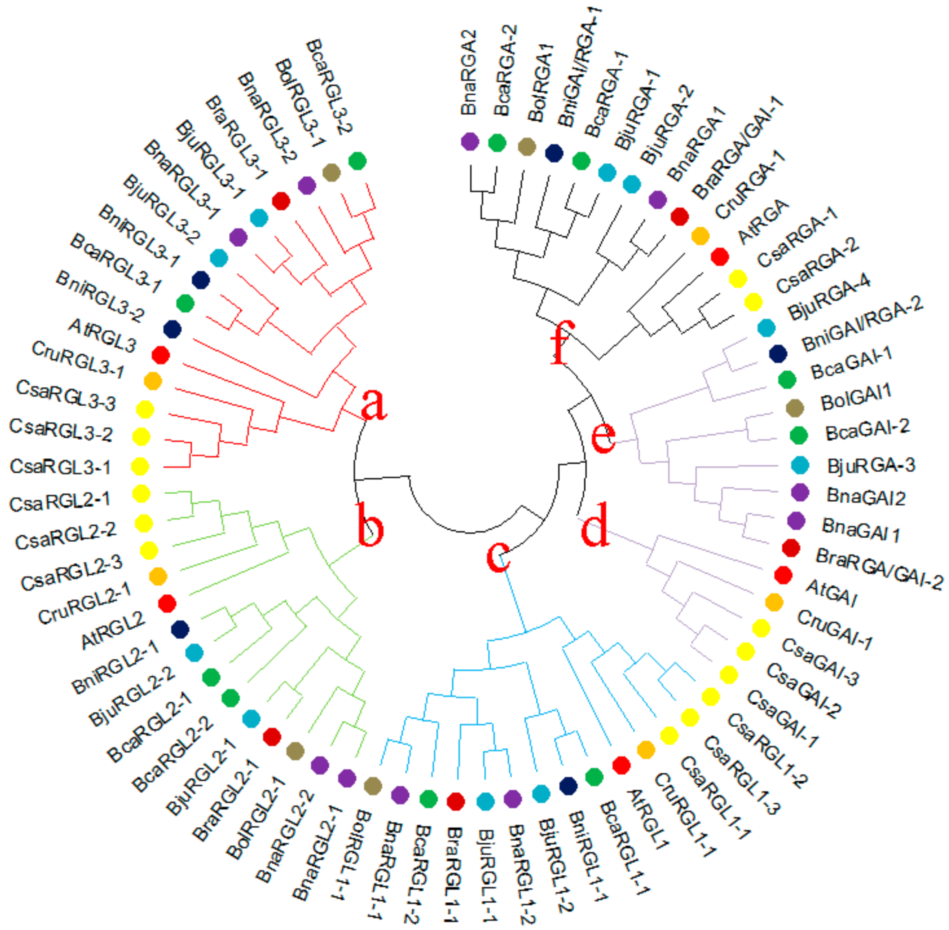

Figure. S4-5 Phylogenetic analysis of Brassicaceae ET-related TFs.

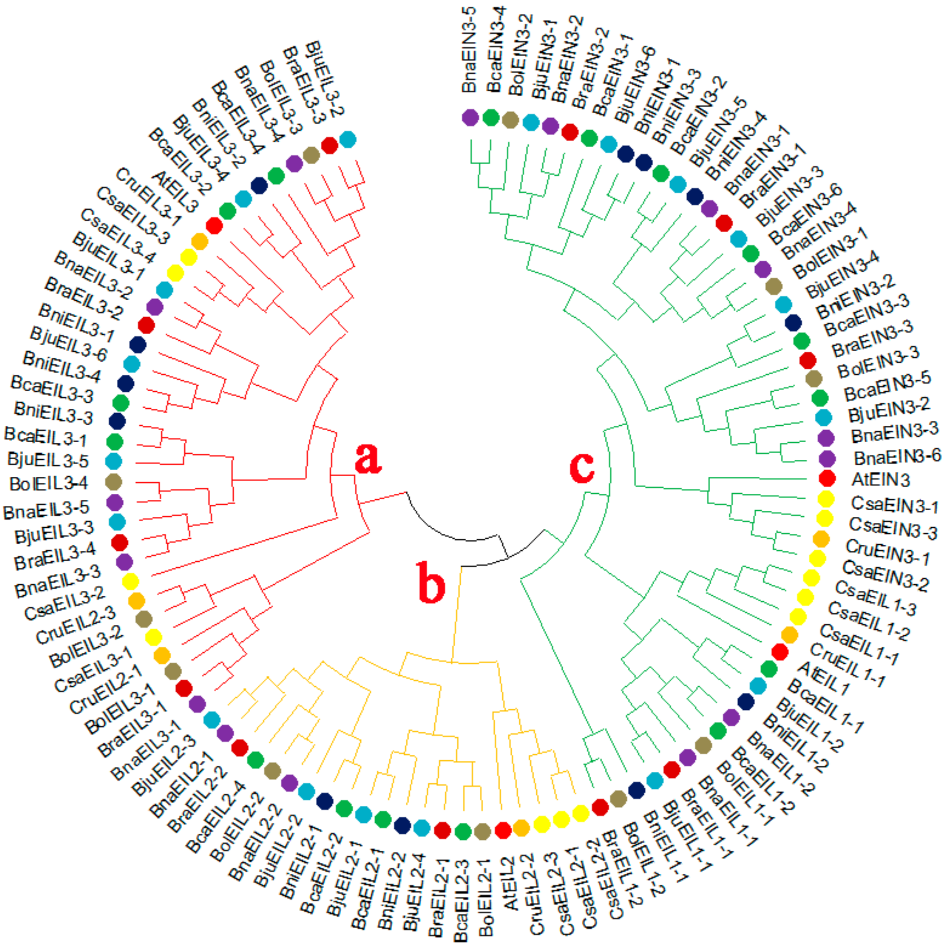

Figure. S4-6 Phylogenetic analysis of Brassicaceae BR-related TFs.

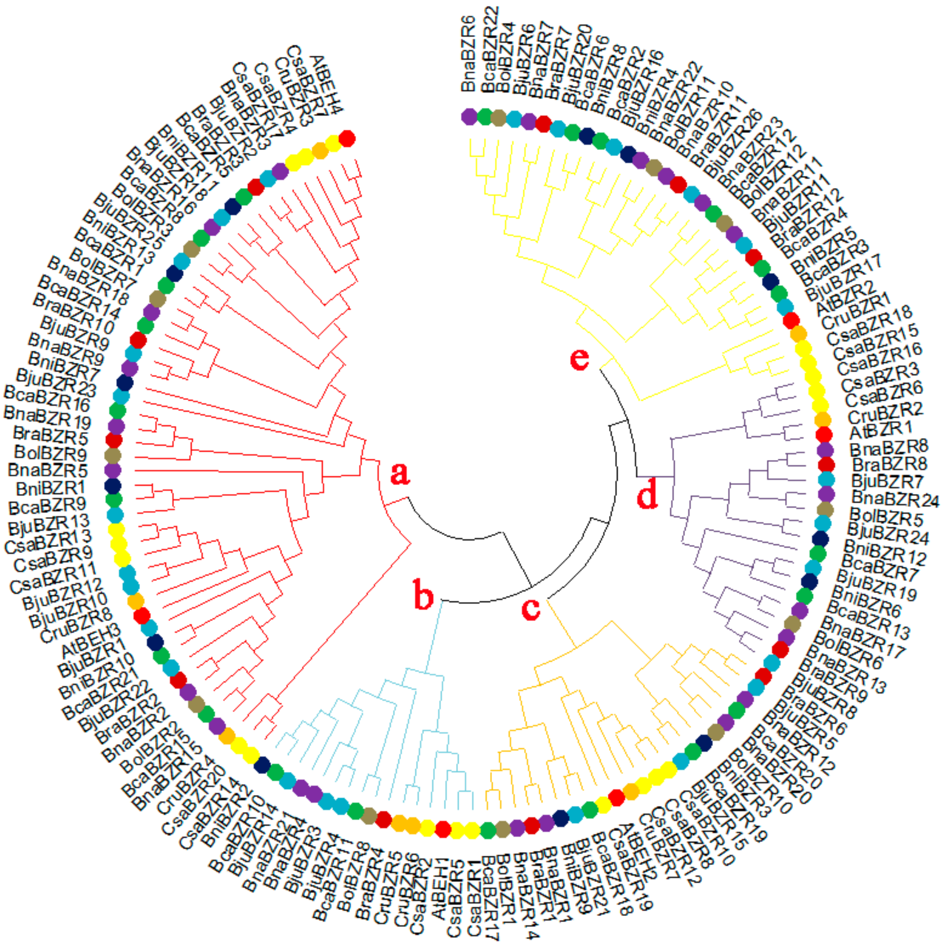

Figure. S4-7 Phylogenetic analysis of Brassicaceae JA-related TFs.

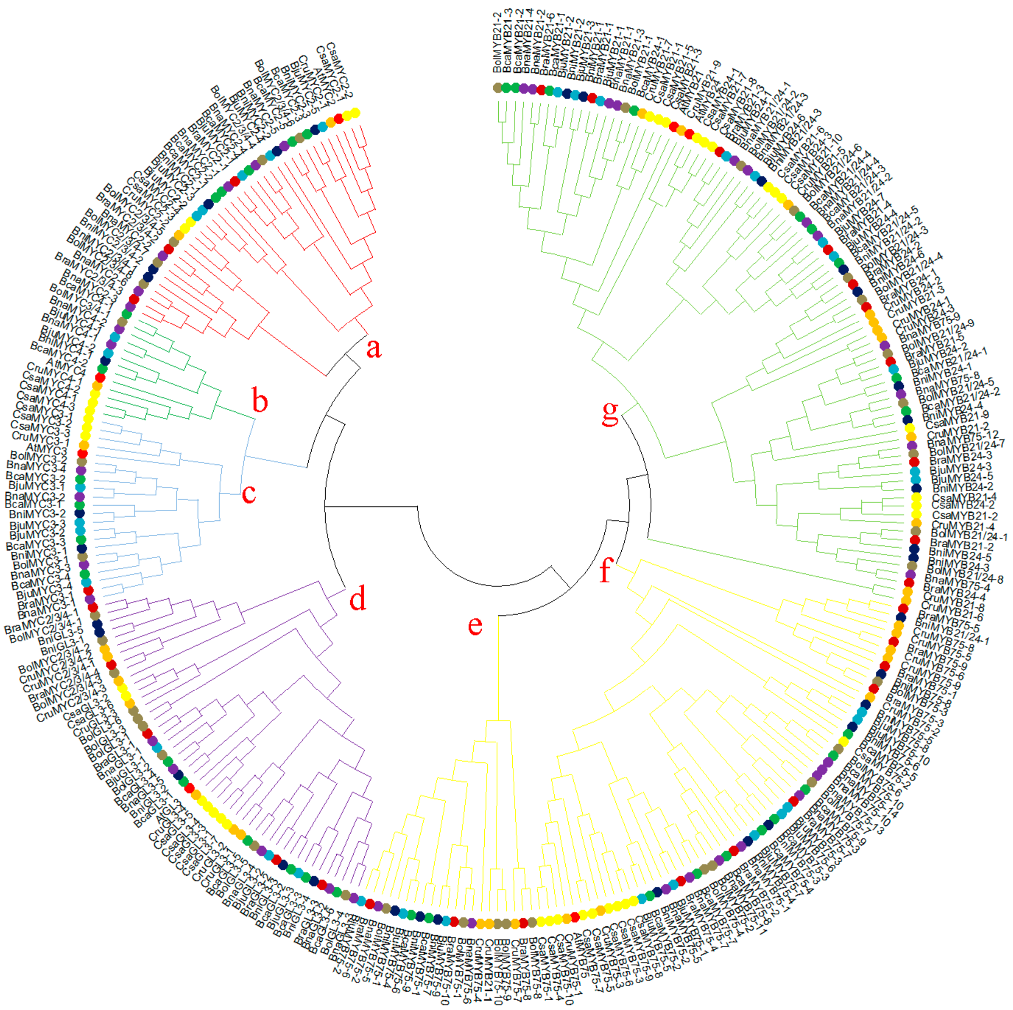

Figure. S4-8 Phylogenetic analysis of Brassicaceae SA-related TFs.

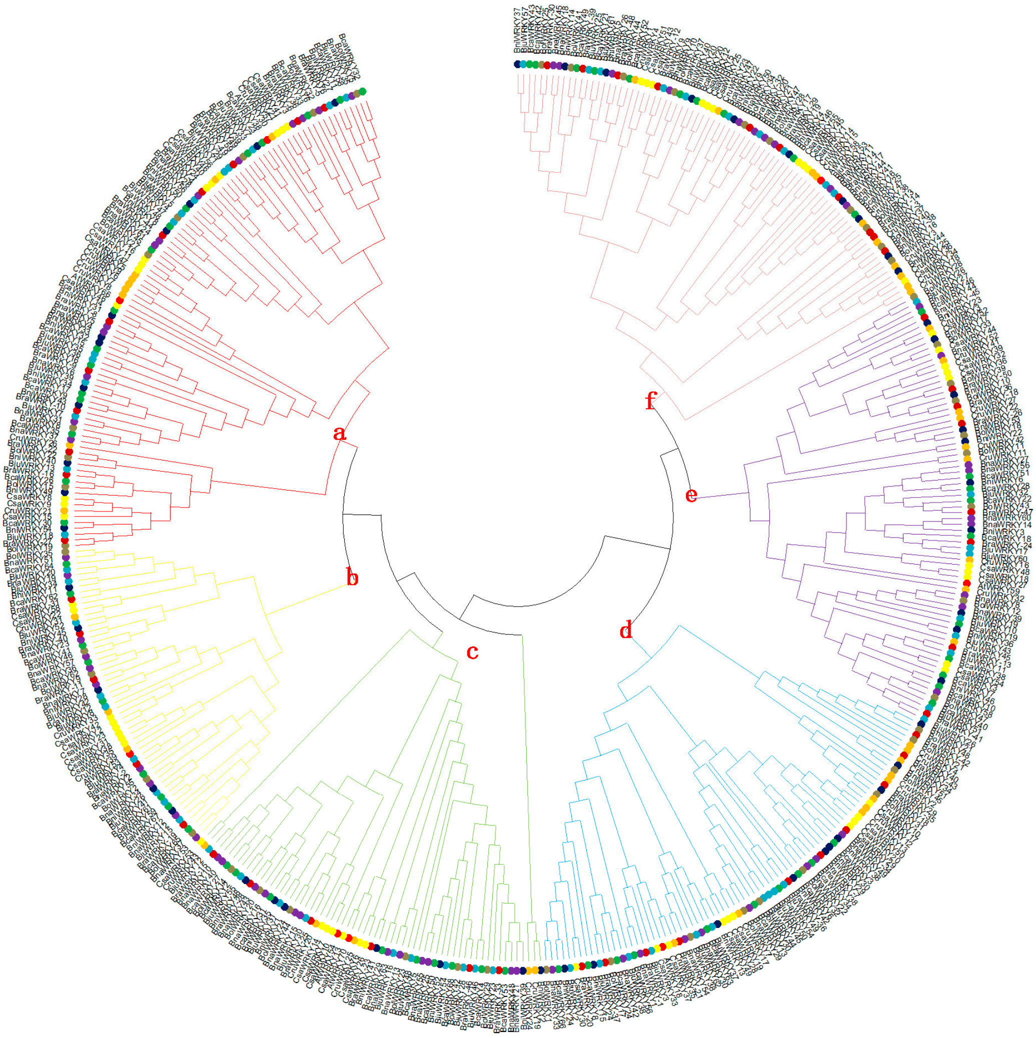

Figure. S4-9 Phylogenetic analysis of Brassicaceae SL-related TFs.

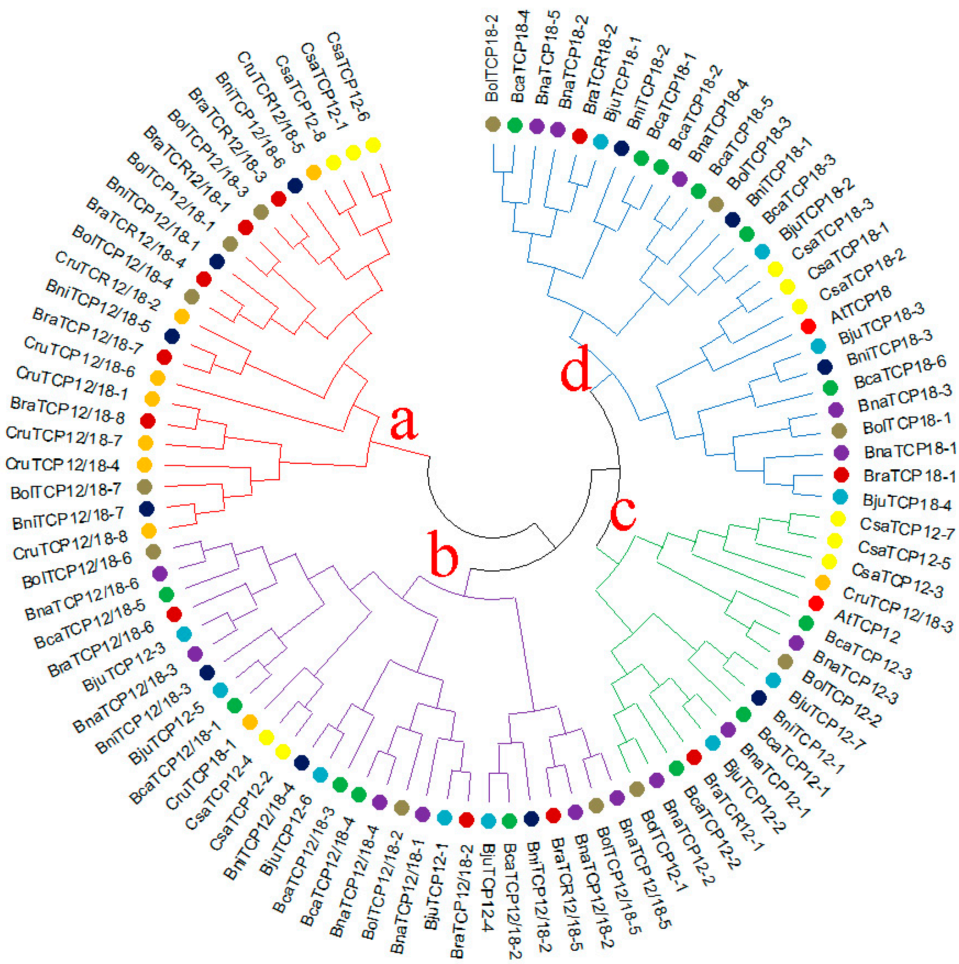

Supplement: Supplementary file 1 [file ijms-23-08762-s001.zip › Figure S4.pdf]
