# Supplementary material for: Genome-Wide Identification of Brassicaceae Hormone-Related Transcription Factors and Their Roles in Stress Adaptation and Plant Height Regulation in Allotetraploid Rapeseed
Source: Int J Mol Sci. 2022 Aug 6;23(15):8762. doi: 10.3390/ijms23158762 (PMC9369146; doi:10.3390/ijms23158762)

Supplemental Figure S5. Synteny of Brassicaceae hormone-related *TFs* in each Brassicaceae species.

Figure. S5-1 Synteny of hormone-related *TFs* in *Arabidopsis*.

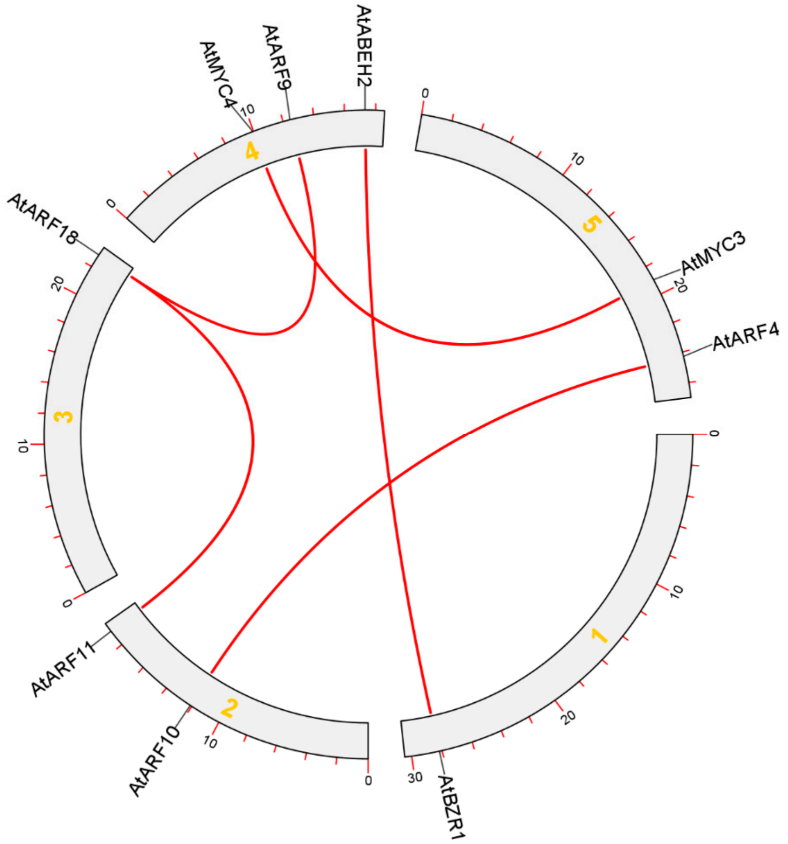

Figure. S5-2 Synteny of hormone-related *TFs* in *Brassica napus*.

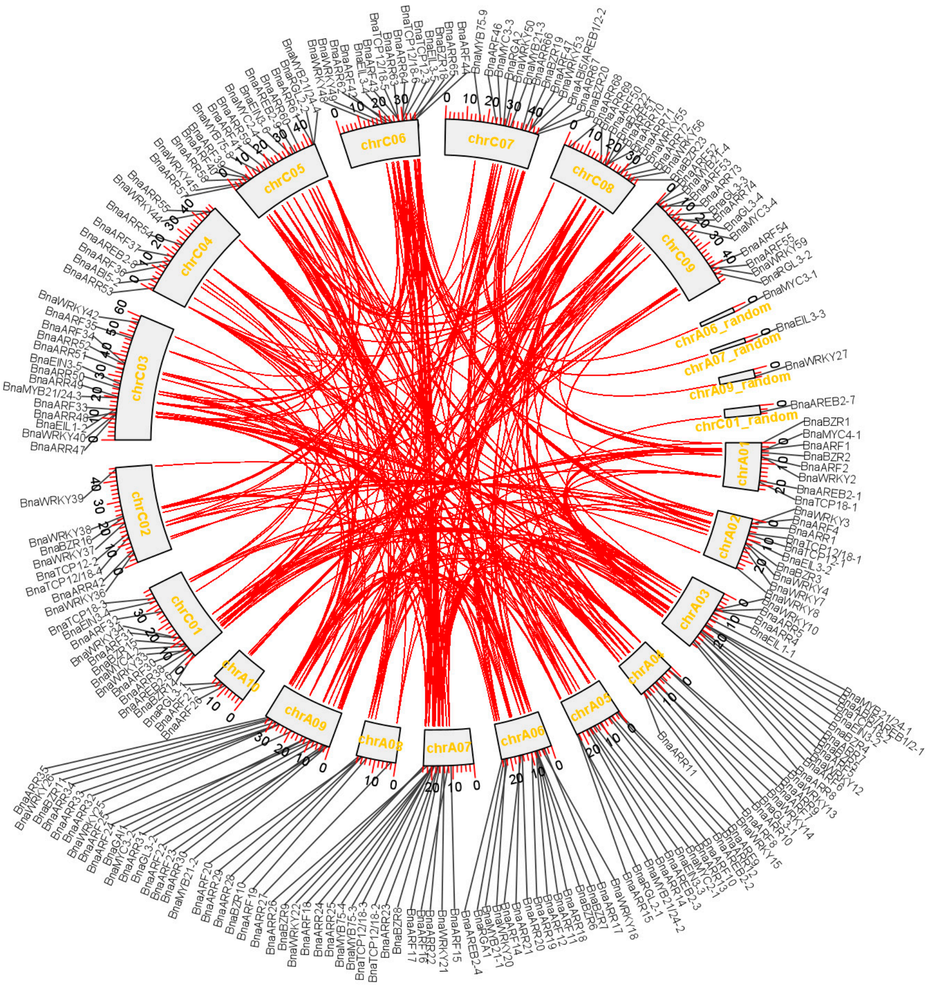

Figure. S5-3 Synteny of hormone-related *TFs* in *Brassica carinata*.

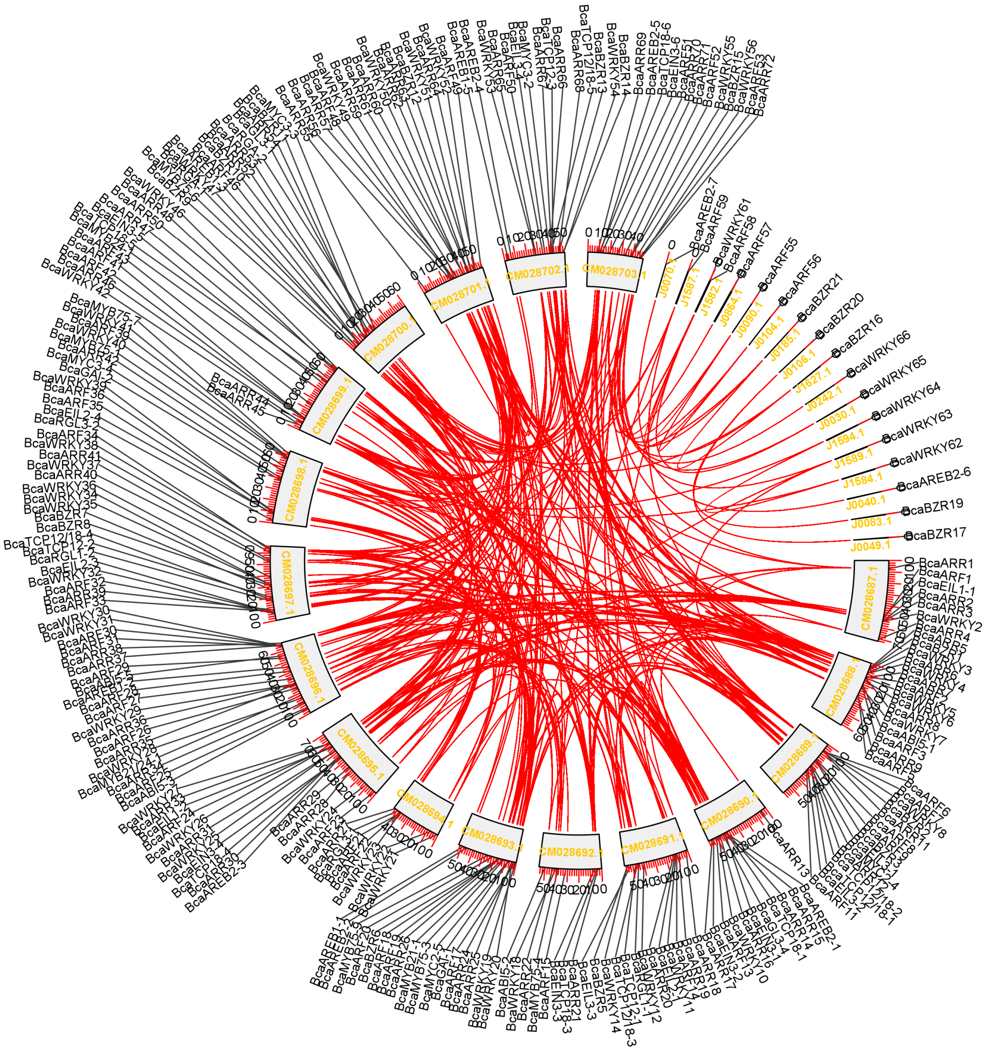

Figure. S5-4 Synteny of hormone-related *TFs* in *Brassica juncea*.

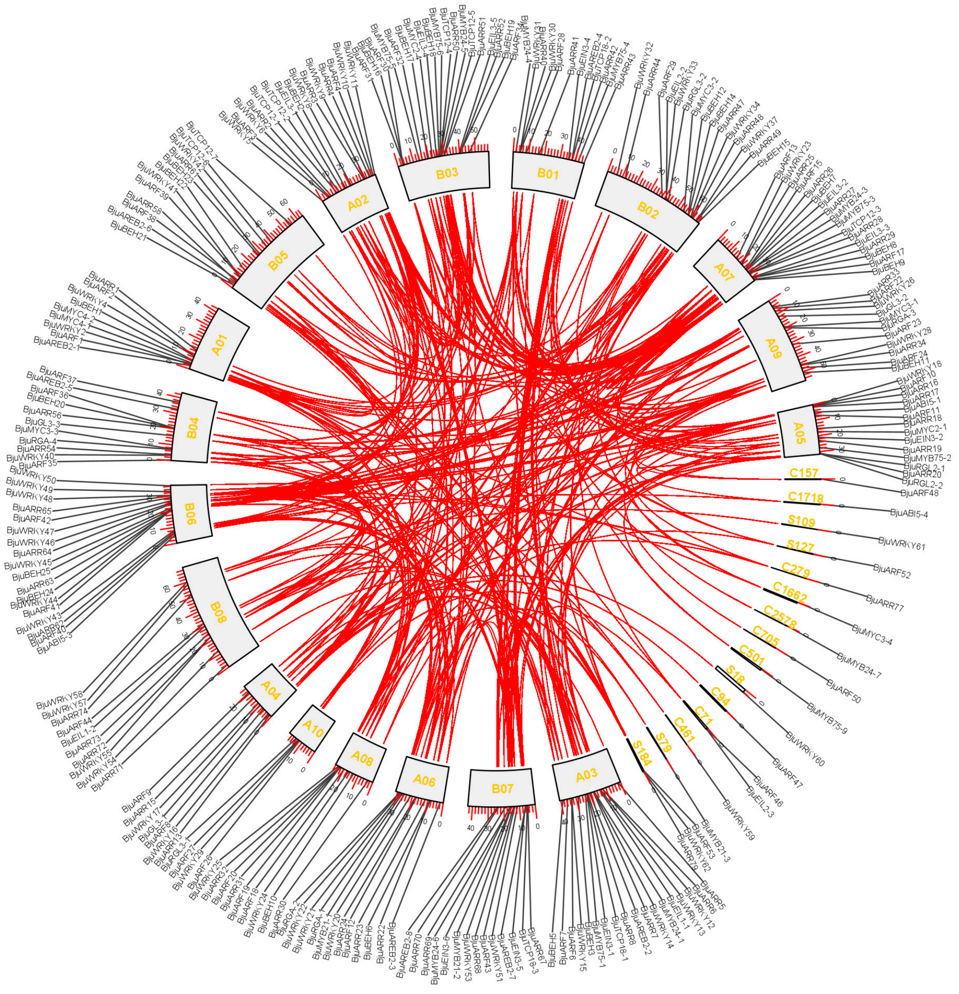

Figure. S5-5 Synteny of hormone-related *TFs* in *Brassica nigra*.

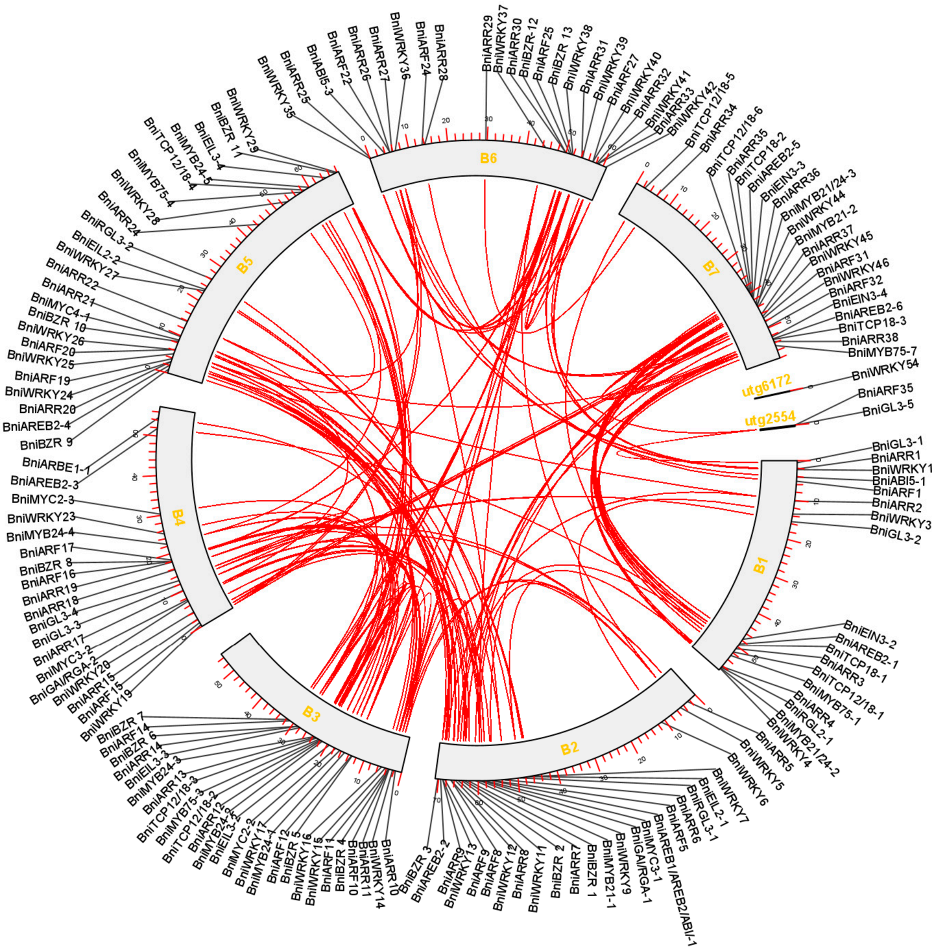

Figure. S5-6 Synteny of hormone-related *TFs* in *Brassica oleracea*.

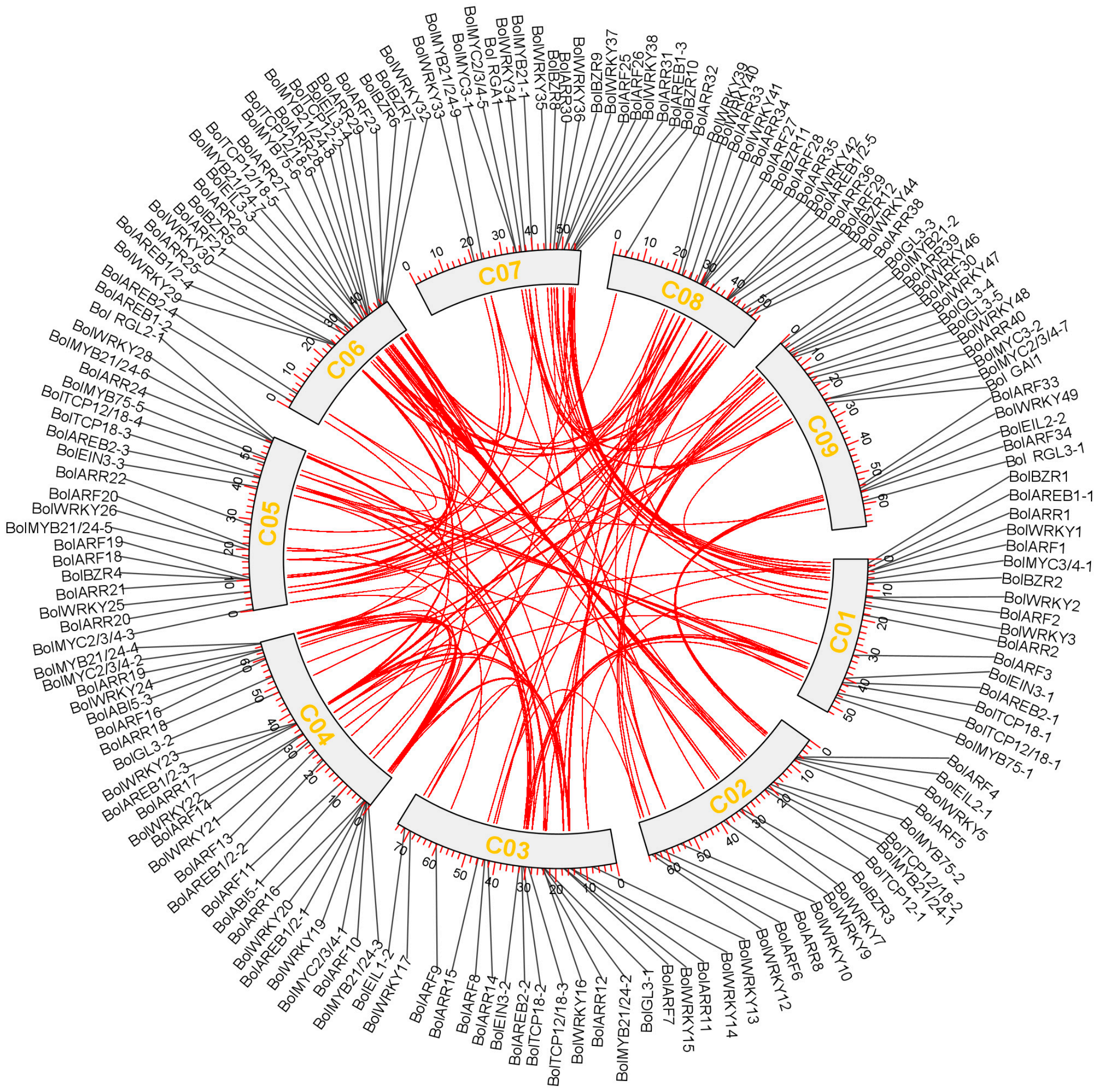

Figure. S5-7 Synteny of hormone-related *TFs* in *Brassica rapa*.

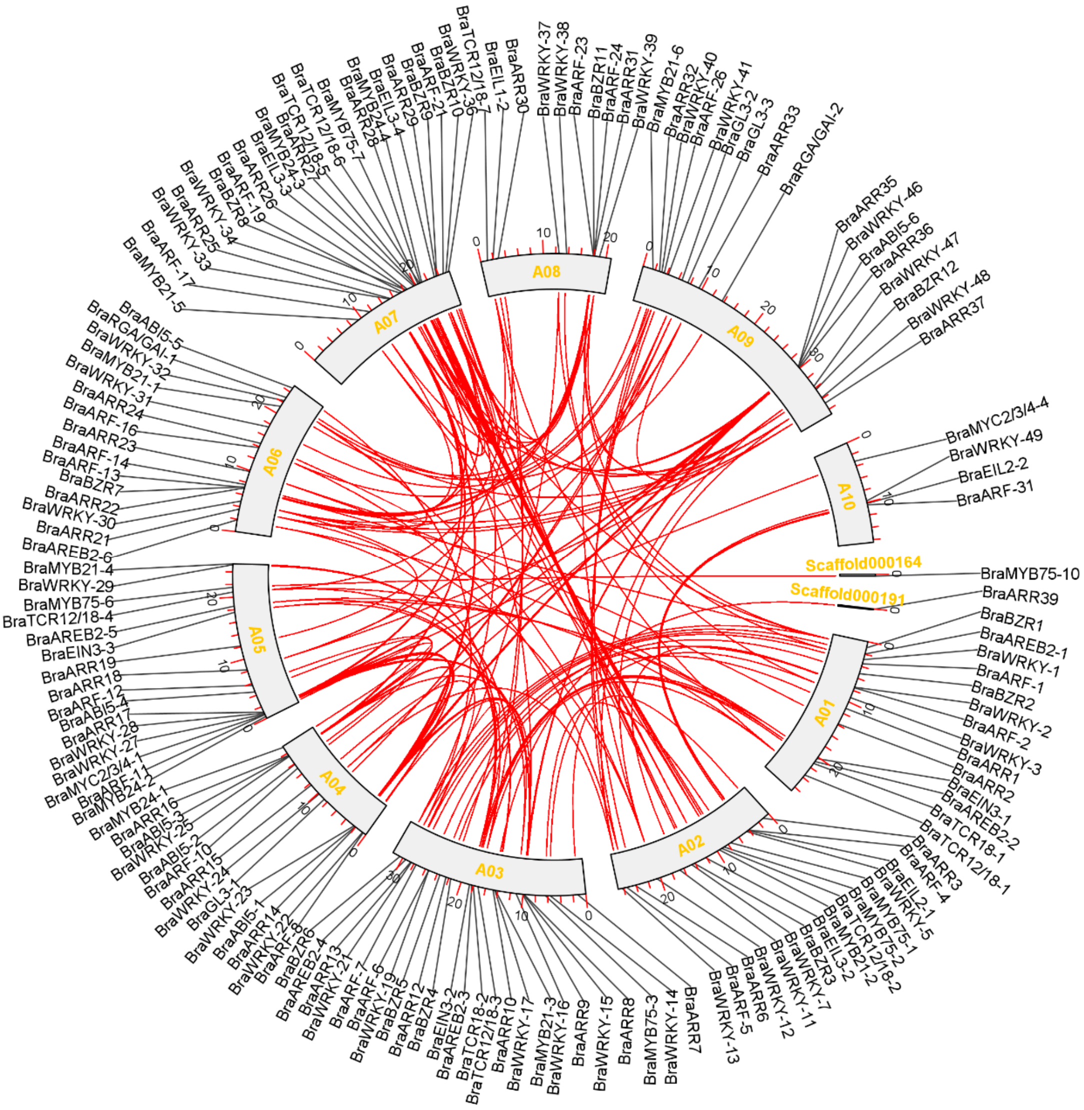

Figure. S5-8 Synteny of hormone-related *TFs* in *Camelina sativa*.

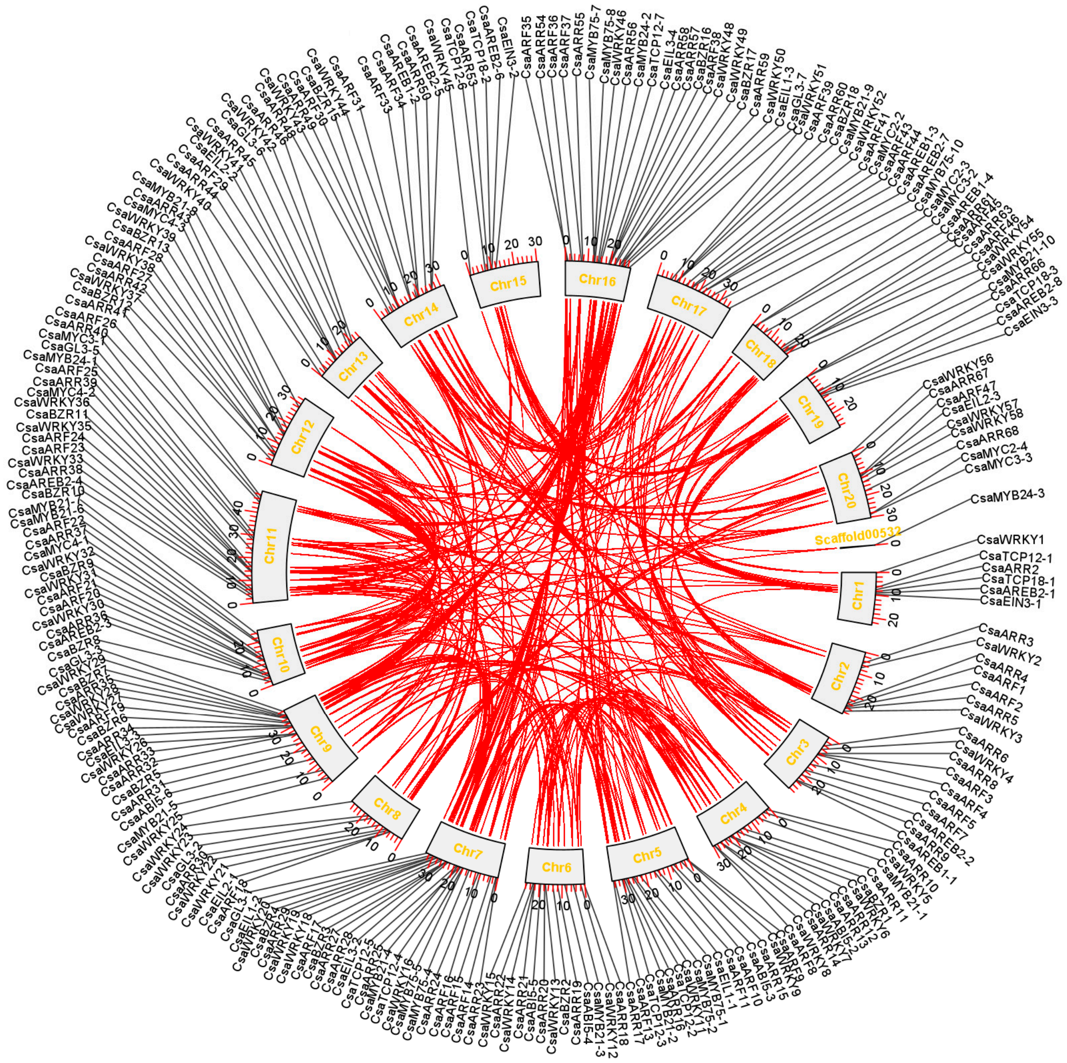

Figure. S5-9 Synteny of hormone-related *TFs* in *Capsella rubella*.

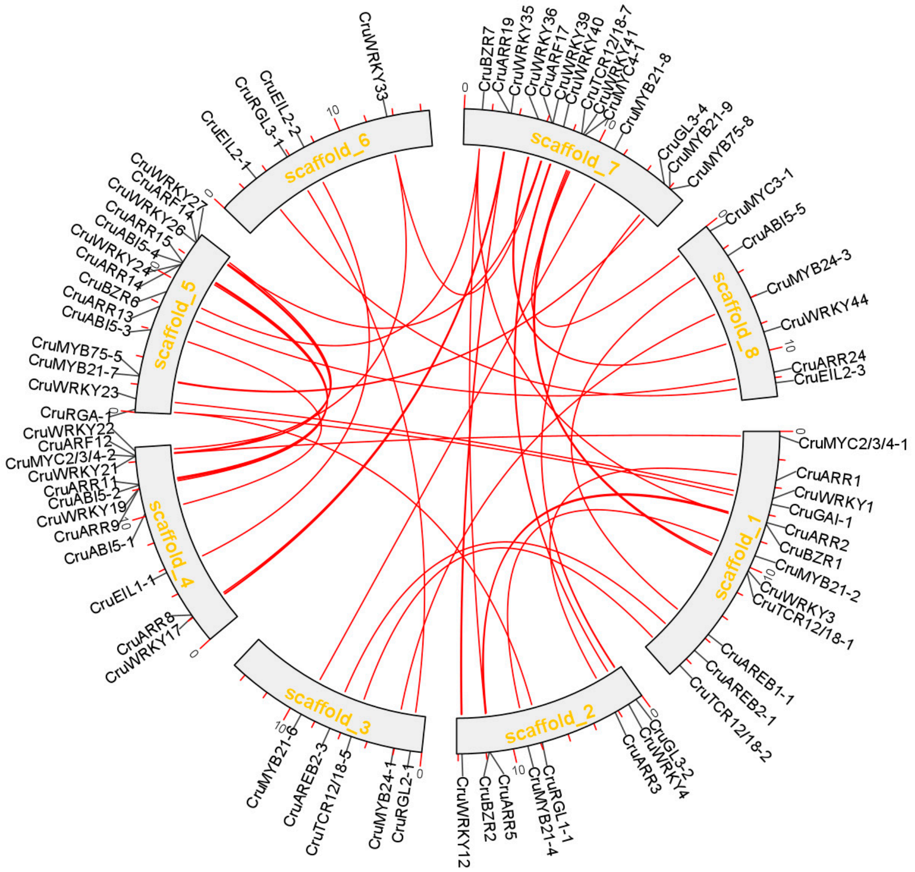

Supplement: Supplementary file 1 [file ijms-23-08762-s001.zip › Figure S5.pdf]
