# Supplementary material for: Genome-Wide Identification of Brassicaceae Hormone-Related Transcription Factors and Their Roles in Stress Adaptation and Plant Height Regulation in Allotetraploid Rapeseed
Source: Int J Mol Sci. 2022 Aug 6;23(15):8762. doi: 10.3390/ijms23158762 (PMC9369146; doi:10.3390/ijms23158762)

Supplemental Figure S6. Synteny of hormone-related *TFs* between *Arabidopsis* and other Brassicaceae species.

Figure. S6-1 Synteny of hormone-related *TFs* between *Arabidopsis* and *Brassica napus.*

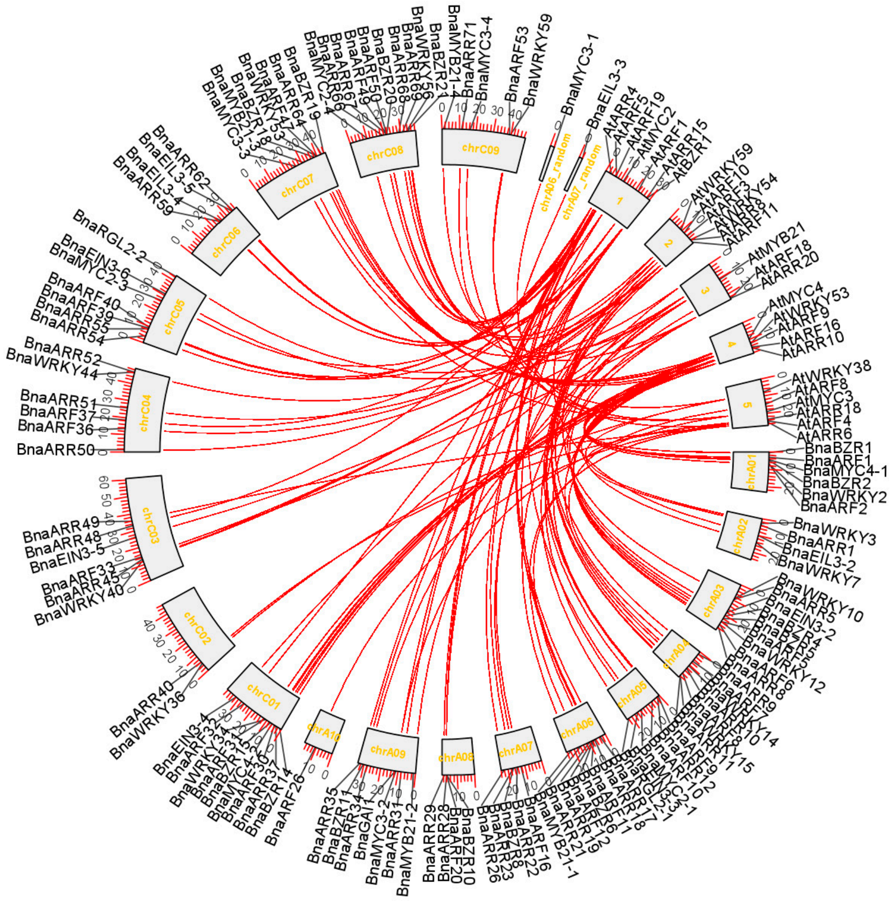

Figure. S6-2 Synteny of hormone-related *TFs* between *Arabidopsis* and *Brassica carinata*.

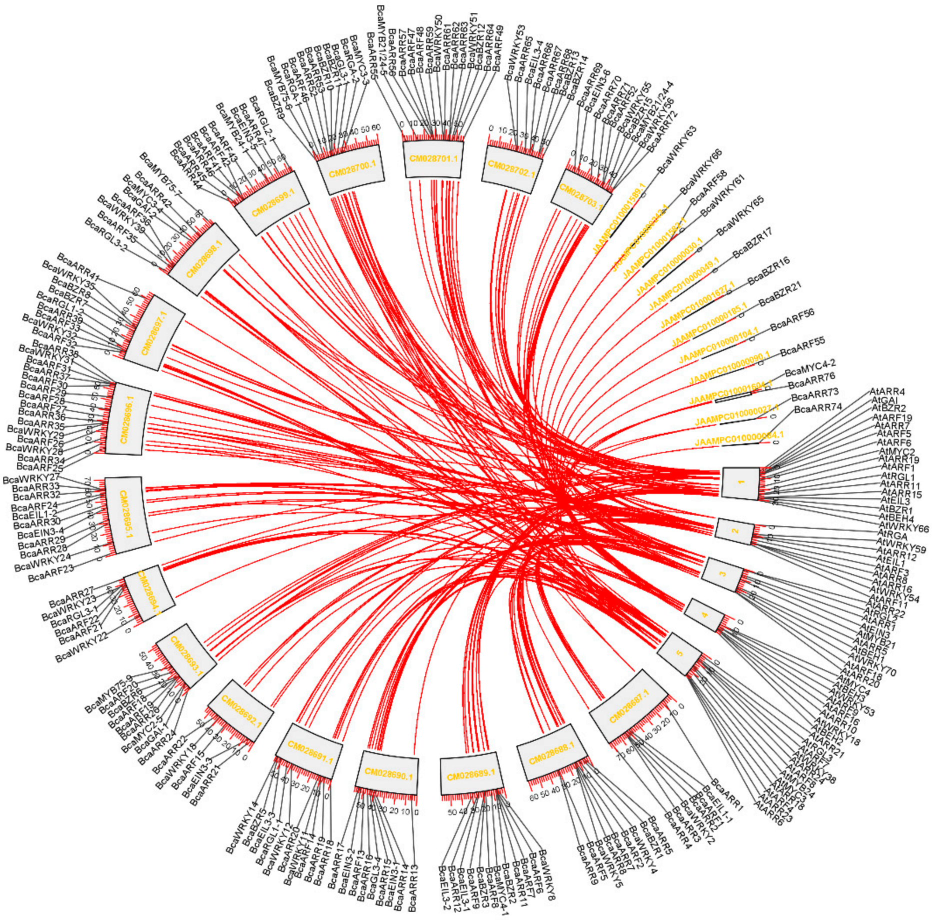

Figure. S6-3 Synteny of hormone-related *TFs* between *Arabidopsis* and *Brassica juncea*.

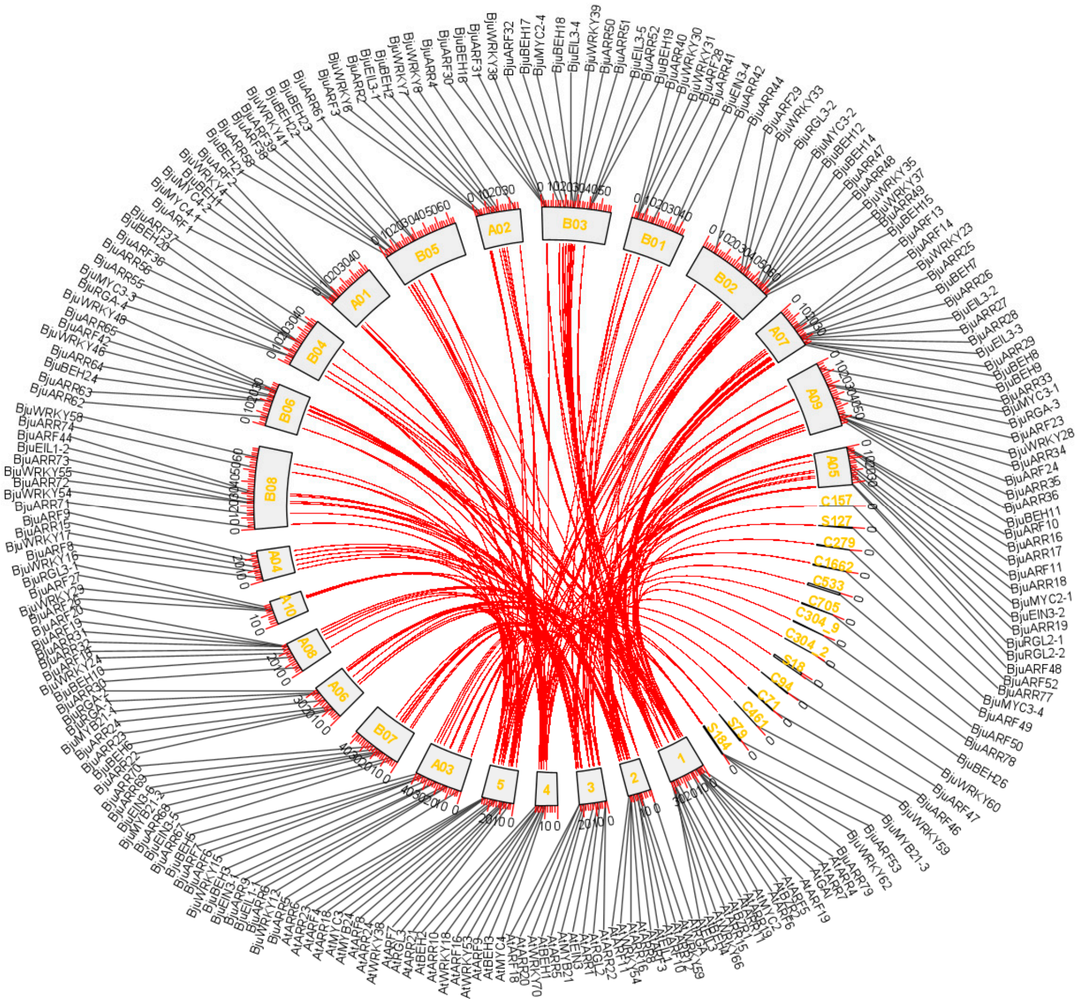

Figure. S6-4 Synteny of hormone-related *TFs* between *Arabidopsis* and *Brassica nigra*.

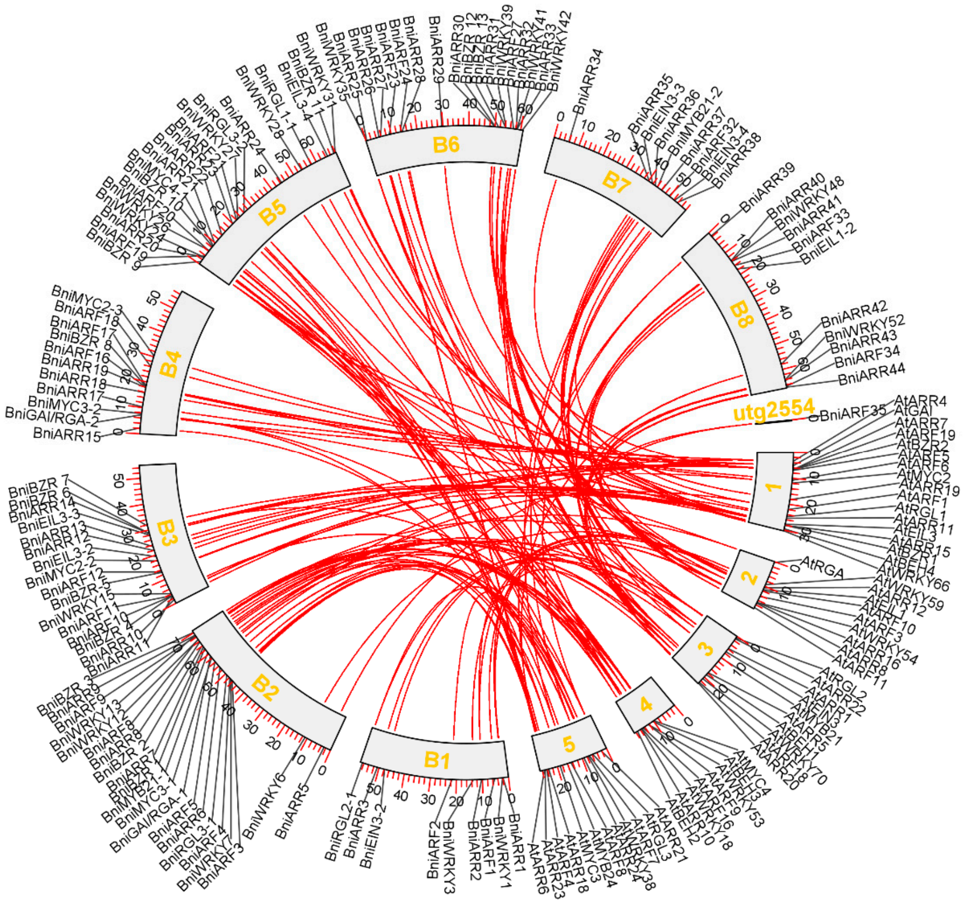

Figure. S6-5 Synteny of hormone-related *TFs* between *Arabidopsis* and *Brassica oleracea.*

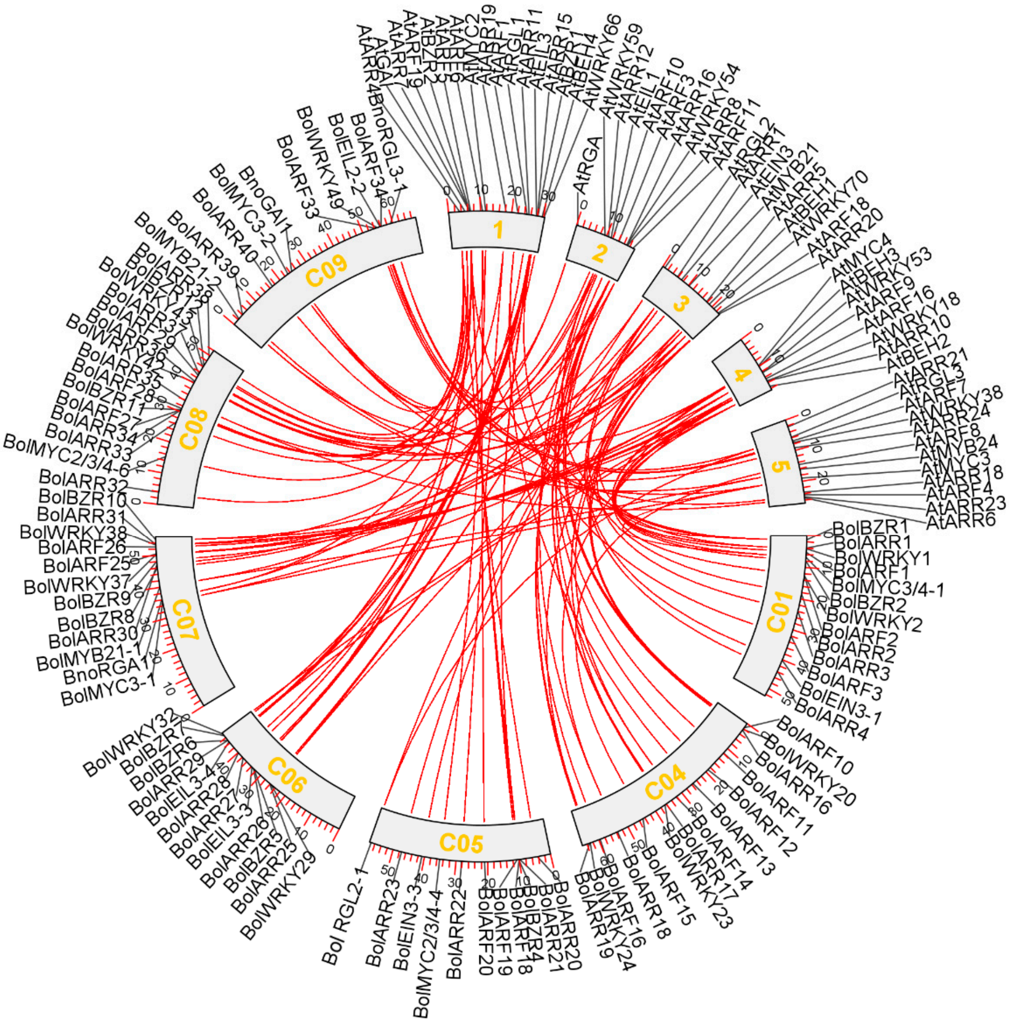

Figure. S6-6 Synteny of hormone-related *TFs* between *Arabidopsis* and *Brassica rapa.*

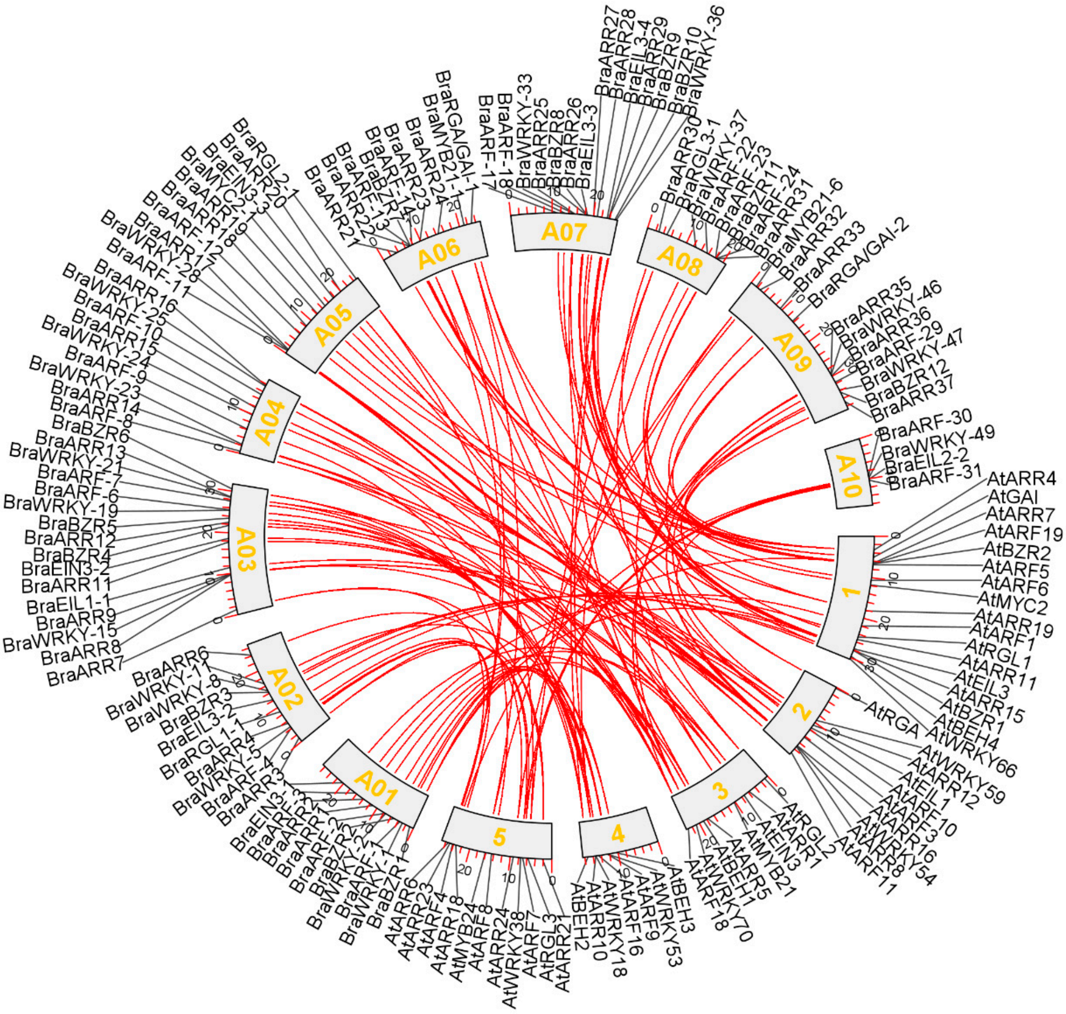

Figure. S6-7 Synteny of hormone-related *TFs* between *Arabidopsis* and *Camelina sativa.*

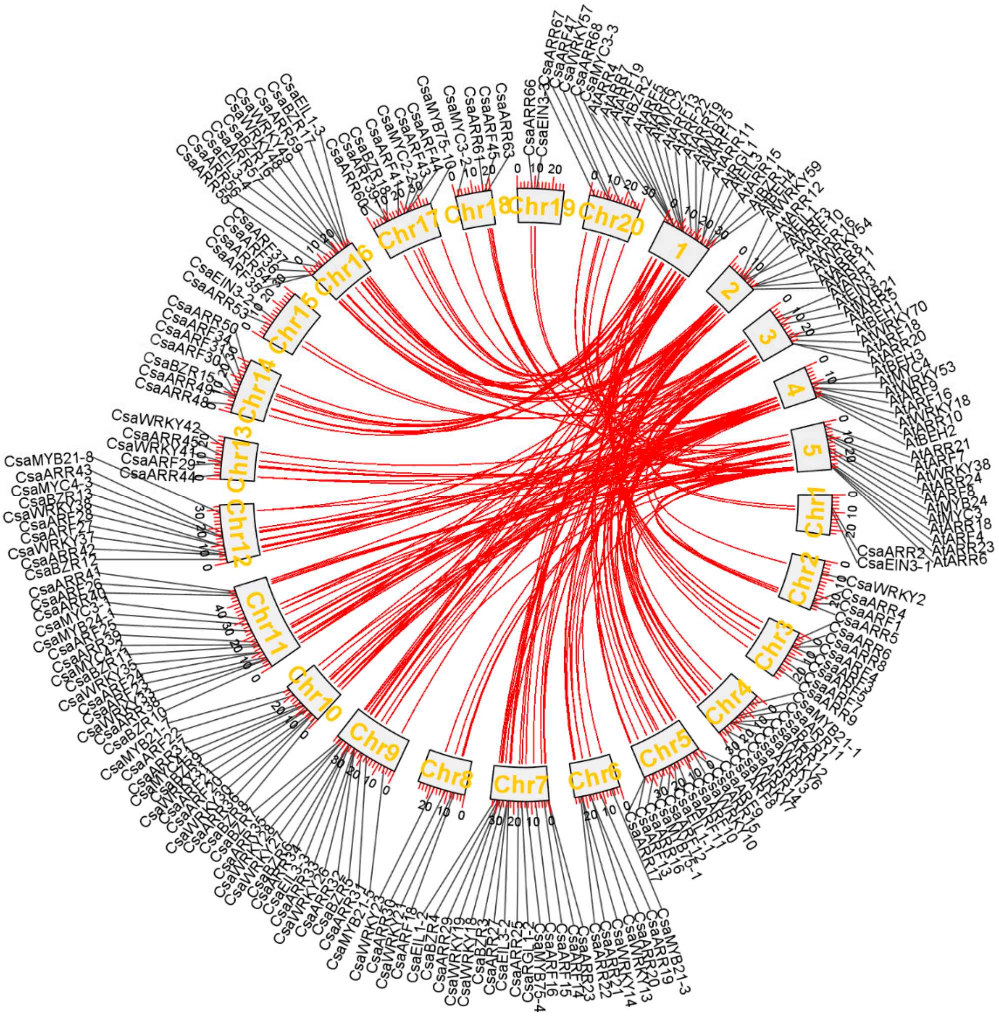

Figure. S6-8 Synteny of hormone-related *TFs* between *Arabidopsis* and *Capsella rubella.*

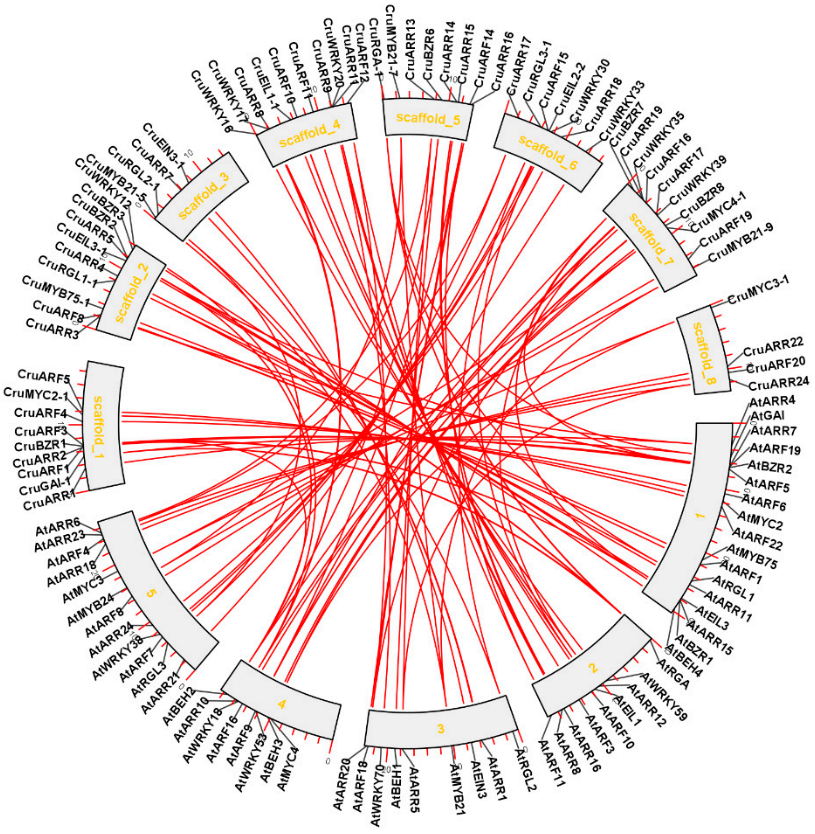

Supplement: Supplementary file 1 [file ijms-23-08762-s001.zip › Figure S6.pdf]
