# Supplementary material for: Genome-Wide Identification of Brassicaceae Hormone-Related Transcription Factors and Their Roles in Stress Adaptation and Plant Height Regulation in Allotetraploid Rapeseed
Source: Int J Mol Sci. 2022 Aug 6;23(15):8762. doi: 10.3390/ijms23158762 (PMC9369146; doi:10.3390/ijms23158762)
Supplement: Supplementary file 1 [file ijms-23-08762-s001.zip › Figure S7.pdf]

Supplemental Figure S7. Expression profiles of *B. napus* hormone-related *TFs* in PH regulation.

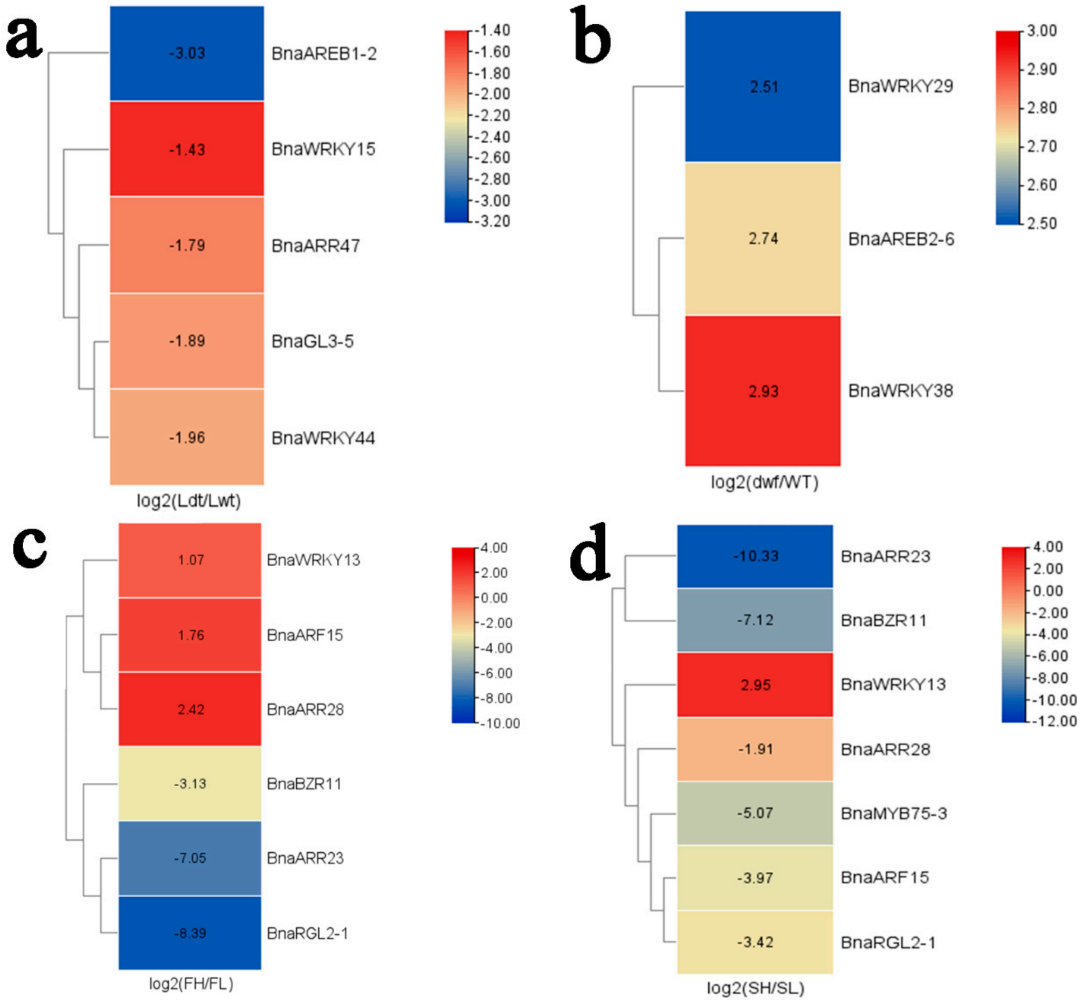

a Expression analysis of hormone-related *TFs* between dwarf (Ldt) and wild type (Lwt) rapeseed. **b** Expression analysis of hormone-related *TFs* between dwarf (dwf) and wild type (WT). **c** Expression analysis of hormone-related *TFs* between FH and FL. **d** Expression analysis of hormone-related *TF* genes between SH and SL.
